# Genetic basis of thiaminase I activity in a vertebrate, zebrafish *Danio rerio*

**DOI:** 10.1038/s41598-023-27612-5

**Published:** 2023-01-13

**Authors:** Catherine A. Richter, Allison N. Evans, Scott A. Heppell, James L. Zajicek, Donald E. Tillitt

**Affiliations:** 1 U.S. Geological Survey, Columbia Environmental Research Center, Columbia, MO 65201 USA; 2 Department of Fisheries, Wildlife, and Conservation Sciences, Oregon State University, Corvallis, OR 97331 USA; 3 Present Address: Department of Microbiology, Oregon State University, Corvallis, OR 97331 USA

**Keywords:** Enzymes, Freshwater ecology, Environmental sciences

## Abstract

Thiamine (vitamin B_1_) metabolism is an important driver of human and animal health and ecological functioning. Some organisms, including species of ferns, mollusks, and fish, contain thiamine-degrading enzymes known as thiaminases, and consumption of these organisms can lead to thiamine deficiency in the consumer. Consumption of fish containing thiaminase has led to elevated mortality and recruitment failure in farmed animals and wild salmonine populations around the world. In the North American Great Lakes, consumption of the non-native prey fish alewife (*Alosa pseudoharengus*) by native lake trout (*Salvelinus namaycush*) led to thiamine deficiency in the trout, contributed to elevated fry mortality, and impeded natural population recruitment. Several thiaminases have been genetically characterized in bacteria and unicellular eukaryotes, and the source of thiaminase in multicellular organisms has been hypothesized to be gut microflora. In an unexpected discovery, we identified thiaminase I genes in zebrafish (*Danio rerio*) with homology to bacterial tenA thiaminase II. The biochemical activity of zebrafish thiaminase I (GenBank NP_001314821.1) was confirmed in a recombinant system. Genes homologous to the zebrafish tenA-like thiaminase I were identified in many animals, including common carp (*Cyprinus carpio*), zebra mussel (*Dreissena polymorpha*) and alewife. Thus, the source of thiaminase I in alewife impacting lake trout populations is likely to be de novo synthesis.

## Introduction

Thiamine (vitamin B_1_) is required by virtually all living organisms^1^. Produced by certain plants, fungi, and bacteria, thiamine is a dietary requirement for all multicellular animals. Loss of sufficient dietary thiamine leads to neurological deficits, cardiac dysfunction, and numerous other physiological outcomes in thiamine deficient individuals. Symptomology of thiamine deficiency in humans most commonly occurs from malnutrition or chronic alcoholism and includes beriberi and Wernicke’s encephalopathy, respectively^2^. Populations of fish and wildlife have experienced declines in numbers and, in certain cases, complete reproductive failures over large geographic areas from thiamine deficiency. Collapse of lake trout (*Salvelinus namaycush*) populations in the Great Lakes of North America^3^, loss of Atlantic salmon (*Salmo salar*) spawning runs in Baltic Sea tributaries in Sweden and Finland^4^, and widespread declines in Northern European bird populations^5,6^ have all been linked to thiamine deficiency. Most recently, populations of Chinook salmon (*Oncorhynchus tshawytscha*) from the Central Valley of California, North America have symptoms of thiamine deficiency^7^. Indeed, these observations have led to the recognition of thiamine deficiency as an emerging issue affecting global biodiversity^8^.

Thiamine is a required co-factor for enzymes in essential metabolic pathways, including energy metabolism and amino acid synthesis. Thiamine deficiency syndromes have been observed in livestock and captive animals that are fed a homogenous diet and in wildlife under conditions of human-influenced ecological changes^4,6,7^. Thiamine synthesis, degradation, and salvage pathways are found in some bacteria, unicellular animals, and plants. Thiamine auxotrophs, including all known multicellular animals, must acquire thiamine through their diets or from bacterial symbionts. Competition for thiamine has resulted in evolution of diverse strategies for thiamine synthesis, uptake, salvage, and degradation^9–11^. However, the mechanisms and consequences of thiamine ecology are not well understood^12^.

Lake trout populations have declined substantially since the 1950s in the Laurentian Great Lakes of North America, and rehabilitation of these populations continues to be a management goal^13^. Recruitment failure of lake trout in the Great Lakes likely results from a combination of factors, including poor survival of early life stages caused by Thiamine Deficiency Complex (TDC)^14,15^. Thiamine deficiency occurs in lake trout because adults ingest prey fish (notably alewife, *Alosa pseudoharengus*, and rainbow smelt, *Osmerus mordax*) that contain high levels of thiaminase, a thiaminolytic enzyme^16^. A laboratory feeding study confirmed that lake trout eating an experimental diet of 100% alewife for two years produced fry with early life stage mortality resulting from thiamine deficiency^17^. Field observations have shown an inverse relation between alewife populations and egg thiamine concentrations in lake trout from Lake Michigan and Lake Huron, and documented natural reproduction of lake trout in Lake Huron following an alewife population crash^18^. Thiaminase enzymes are known to be produced by bacteria^19,20^ and one eukaryote, the single-celled amoeboflagellate *Naegleria gruberi*^21^. Thiaminases cleave thiamine into pyrimidine and thiazole ring components with either an organic nucleophile co-substrate (thiaminase I, EC 2.5.1.2), or water (thiaminase II, EC 3.5.99.2).

The source of the thiaminase I enzyme activity observed in fishes is currently undefined. A bacterial source of thiaminase I in fish has been supported by circumstantial evidence for the involvement of the thiaminase I-producing bacteria *Paenibacillus thiaminolyticus*^17,22^. However, quantitative analysis of populations of *P. thiaminolyticus* in alewife viscera did not support a substantial role for this bacteria species as the source of the thiaminase I activity observed in alewife^23^. Furthermore, in an evaluation of factors affecting the presence of thiaminase I activity in Great Lakes fishes, diet was not a strong predictor^24^. An investigation of pH optima of thiaminases from *P. thiaminolyticus* and several species of fish showed that pH optima varied among the fish examined, and only two of the four fish species had pH optima similar to *P. thiaminolyticus*^25^. Protein chemistry investigations provided additional evidence for de novo synthesis of thiaminase I by fish. Partial purifications of thiaminase I enzymes from bacteria, fish, and a variety of crustaceans suggested that thiaminase I biochemical properties, including molecular mass, substrate specificity, and co-substrate specificity, varied depending on the source^26,27^. Fish and shellfish thiaminase I were more different from bacterial thiaminase I than from each other, and thiaminase I from different bacteria were usually more similar to one another than to thiaminase I of other taxa^26,27^. Peptide fragments have been sequenced for common carp (*Cyprinus carpio*) and red cornetfish (*Fistularia petimba*) thiaminase I, and neither showed significant sequence similarity to bacterial enzymes^27,28^. Taken together, the protein chemistry and sequence evidence suggest that thiaminase I enzymes from fish are different from bacterial thiaminase I and are possibly produced de novo.

The previous study of thiaminase I activity in red cornetfish suggested a possible path to identify putative thiaminases that are active in fish tissues by protein sequence homology. The authors collected liver tissue from red cornetfish, isolated a soluble protein homogenate, and conducted a series of protein fractionations via isoelectric focusing and gel filtration while monitoring thiaminase I activity with an in-gel assay^27^. They described a 100 kDa holoenzyme and active subunits of 22 and 25 kDa^27^. The 25 kDa active subunit was purified by 250-fold relative to the initial homogenate, and was subjected to Edman sequencing, producing an 18 amino acid N-terminal sequence, and three internal peptide sequences following trypsin digestion that ranged from 5 to 11 amino acids^27^.

In the present study we used existing thiaminase I protein sequence information from the Nishimune et al. study^27^ to identify candidate genes in fishes that may encode a thiaminase I. In a parallel, complementary approach, we sought to empirically determine biochemical characteristics of thiaminase I enzymes from fishes and assess whether these characteristics were consistent with a de novo source of thiaminase I. We identified putative thiaminase I genes in zebrafish (*Danio rerio*), common carp, and alewife. The empirically measured biochemical properties from thiaminase I extracted from fish tissues differed across species and matched predictions from the candidate gene sequences. We expressed a zebrafish candidate thiaminase I gene in a recombinant bacterial system and confirmed that it encoded a functional thiaminase I enzyme. Together, these lines of evidence support the conclusion that some fish can synthesize their own thiaminase I enzymes de novo.

## Results

**Figure 1:**
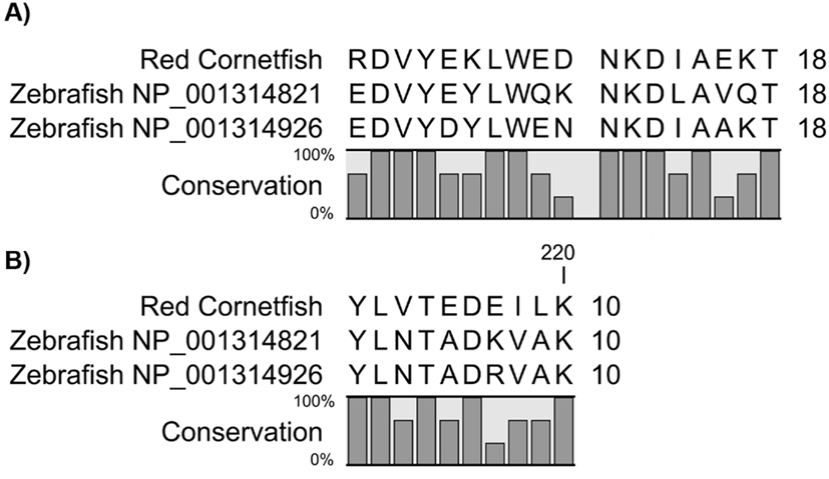
Identification of putative zebrafish (*Danio rerio*) thiaminases. Sequence alignment of previously reported thiaminase I peptide fragments from red cornetfish (*Fistularia* *petimba*) with candidate zebrafish thiaminase I protein sequences. (**A**) N-terminal thiaminase I peptide fragment from red cornetfish aligned with residues 25–40 of zebrafish protein NP_001314821 and residues 39–56 of zebrafish protein NP_001314926. (**B**) Internal thiaminase I peptide fragment from red cornetfish aligned with residues 195–204 of zebrafish protein NP_001314821 and residues 210–219 of zebrafish protein NP_001314926.

We identified candidate thiaminase I genes in zebrafish based on sequence homology to previously reported thiaminase I peptide fragment sequences from red cornetfish^27^. A BLASTP search of the GenBank nr protein database restricted to zebrafish (taxid:7955) with the red cornetfish N-terminal thiaminase I peptide fragment sequence RDVYEKLWEDNKDIAEKT^27^ showed significant (E-value < 0.05) sequence homology to two paralogous zebrafish proteins, and one internal peptide fragment sequence YLVTEDEILK^27^ also aligned to the same zebrafish proteins (Fig. 1). The other two internal peptide fragment sequences identified from red cornetfish thiaminase I, FHMEGVSEIIP and DRFES^27^, could not be aligned to the zebrafish proteins. The N-terminal fragment had 10/18 (56%) sequence identity among the three sequences (Fig. 1). The internal peptide fragment alignment was not statistically significant but had 5/10 (50%) sequence identity among the three sequences (Fig. 1). The paralogous zebrafish sequences are gene symbol: si:ch1073-67j19.1, ZFIN:ZDB-GENE-141216-297, RefSeq mRNA accession NM_001327892.1, uncharacterized protein LOC100332348 precursor accession NP_001314821.1, and gene symbol: si:ch1073-67j19.2, ZFIN:ZDB-GENE-141216-263, RefSeq mRNA accession NM_001327997.1, uncharacterized protein LOC101882838 precursor accession NP_001314926.1. Both candidate zebrafish thiaminase I genes are represented in the zebrafish reference genome and located on zebrafish chromosome 4 adjacent to each other as a direct repeat. They have similar intron–exon structures typical of eukaryotic genes, with three exons and two introns. Expression of mRNA transcripts of both zebrafish thiaminase I genes is supported by sequence data from cDNA libraries derived from mRNA isolated from zebrafish tissue. The protein sequences have 64% identical amino acids and 77% similar amino acids. Their signal sequences are less conserved than the mature protein sequences. We do not know of protein expression data supporting expression of the zebrafish TenA-like thiaminase I proteins. However, we were able to detect thiaminase I activity in samples of zebrafish tissues, in both in-gel assays (Supplementary Fig. S1) and in a quantitative radiometric assay (Supplementary Table S1)^29^. The thiaminase I activity measured in whole zebrafish homogenates ranged from 19,800 to 44,700 pmol/g/min (Supplementary Table S1).

**Figure 2:**
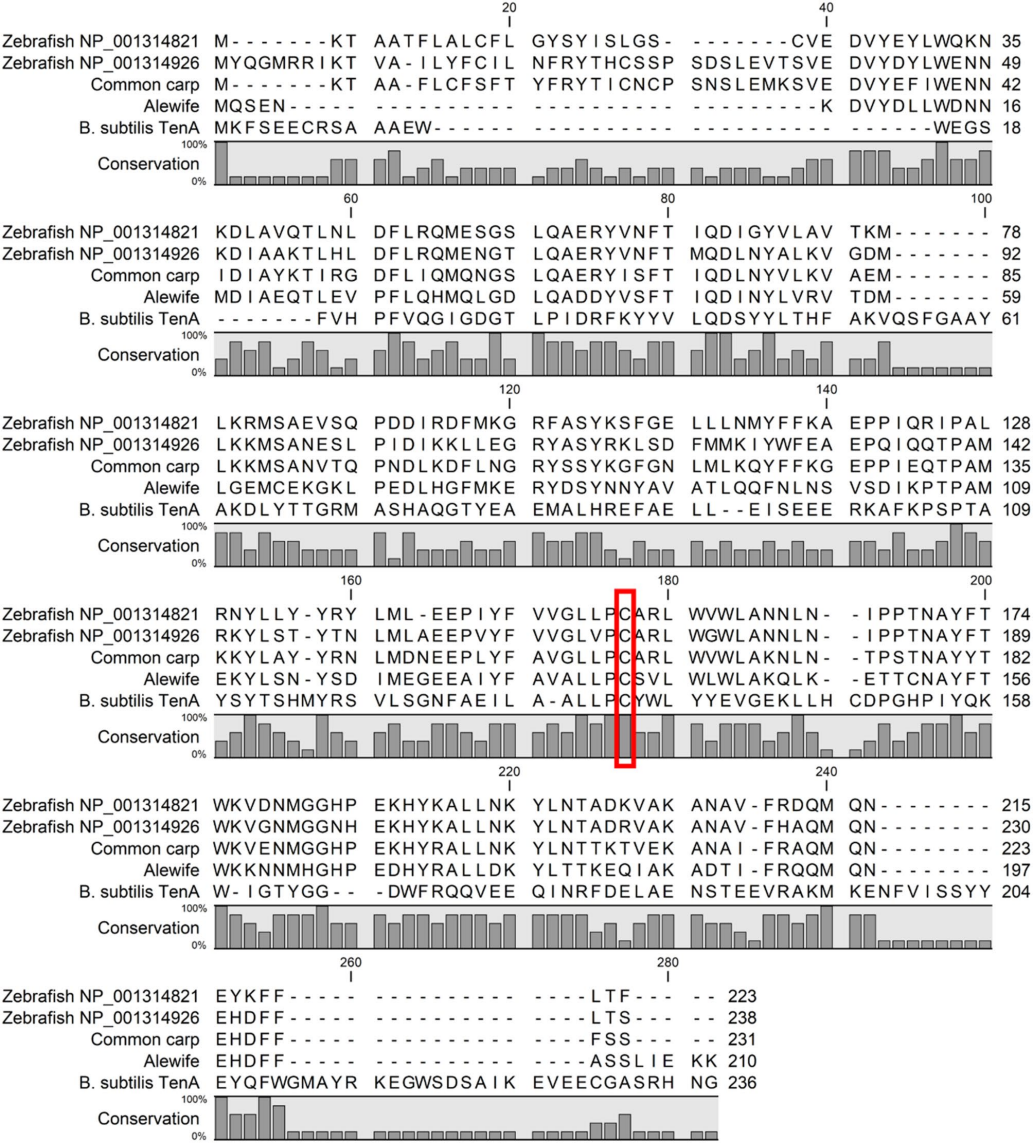
Alignment of known and putative thiaminases. Protein sequence alignment of candidate zebrafish (*Danio rerio*) tenA-like thiaminase I protein sequences with candidate common carp (*Cyprinus carpio*) tenA-like thiaminase I (XP_042594753), candidate alewife (*Alosa pseudoharengus*) tenA-like thiaminase I, and thiaminase II protein TenA (*Bacillus subtilis*, NP_389047). The position of the TenA active site cysteine is marked with a red box.

The candidate zebrafish protein sequence (NP_001314821.1) was then used to search GenBank and transcribed sequences previously reported from alewife^30^ for homologous sequences. A conserved domain search showed homology to the TenA_PqqC-like superfamily (cl38925, E-value 3.61 × 10^–40^), which includes TenA-like proteins including TenA_C and TenA_E proteins, as well as pyrroloquinoline quinone (PQQ) synthesis protein C. The candidate zebrafish thiaminase I protein sequence (NP_001314821.1) had a significant (E-value 0.004) alignment to the well-characterized thiaminase II protein TenA from *Bacillus subtilis* (NP_389047, 22% identity, 42% similarity) that included conservation of the TenA active site Cysteine at a.a. 135 (Fig. 2)^20,31^. Homologous genes were identified from the GenBank nr database in common carp (XP_042594753, 63% identity, 78% similarity, E-value 6 × 10^–105^) and from a previously published transcriptome data set in alewife (Supplementary Fig. S2, 49% identity, 65% similarity, E-value 4 × 10^–71^) (Fig. 2)^30^. The candidate zebrafish thiaminase I genes did not have sequence similarity to known microbial thiaminase I genes such as *P. thiaminolyticus* thiaminase I (WP_087440168)^32^ and *N. gruberi* thiaminase I (4HCW_A)^21^.

A systematic search of the GenBank nr database revealed many homologs to the putative zebrafish TenA-like thiaminase I protein sequence (NP_001314821.1). An unrestricted search of the nr database with a maximum of 100 matches resulted in all 100 matches in bony fish species with scores ranging from 202 to 343 (Supplementary Table S2). A search restricted to Eukaryotes but excluding bony fish resulted in matches with primitive fish, chordates, and diverse invertebrates including bivalves, with scores ranging from 53.5 to 162 (Supplementary Table S3). A search of nr excluding Eukaryotes revealed matches to bacterial TenA proteins, with scores ranging from 60.8 to 90.5 (Supplementary Table S4).

We also investigated an N-terminal peptide fragment previously reported to be associated with thiaminase I activity in common carp^28^. The fragment sequence was identical to residues 30–49 of common carp corticosteroid-binding globulin-like isoform X1 (XP_018935807, now updated to XP_018935808.2), with a predicted molecular mass of 46.7 kDa. A recombinant construct of this protein could not be expressed in soluble form, so its thiaminase I activity could not be tested.

**Figure 3:**
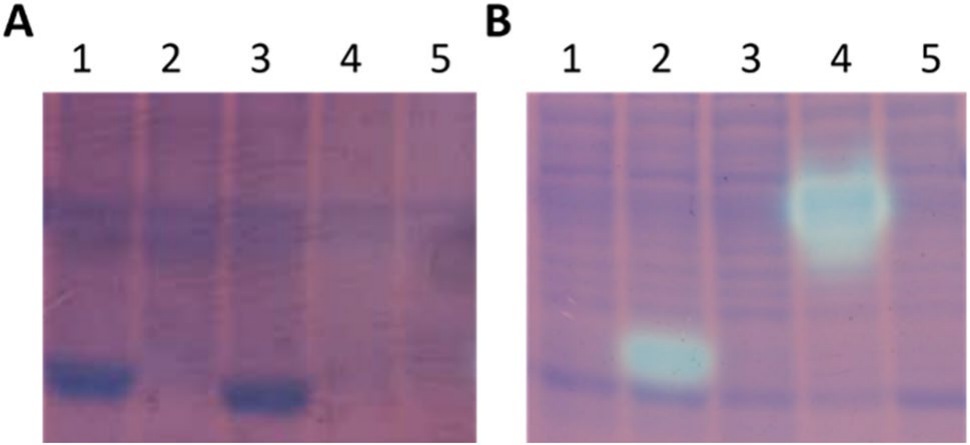
The zebrafish (*Danio rerio*) tenA-like thiaminase I gene encodes a functional thiaminase. Solubility and thiaminase I activity of recombinant candidate thiaminase I genes overexpressed in *Escherichia coli*, stained for activity showing thiamine degradation as clear areas, and with subsequent Coomassie blue protein stain. Gels are cropped to show relevant lanes and mobility range; original gels are presented in Supplementary Fig. 9. Panel A, insoluble fractions; Panel B, soluble fractions; lane 1, common carp (*Cyprinus* *carpio)*, lane 2, zebrafish; lane 3, alewife (*Alosa pseudoharengus*); lane 4, *Paenibacillus thiaminolyticus*; lane 5, empty pET52b vector.

Overexpression of a candidate zebrafish thiaminase I gene (NP_001314821.1) in *Escherichia coli* produced an active thiaminase I enzyme (Fig. 3). The alewife and common carp candidate thiaminase I proteins were expressed in inclusion bodies and could not be renatured to soluble forms. The zebrafish candidate thiaminase I protein was expressed in the soluble fraction of the protein extract and showed thiaminase I activity (Fig. 3) at the expected molecular weight (Supplementary Fig. S3)^33^.

The biochemical characteristics of thiaminase I differed across the species examined, *P. thiaminolyticus*, common carp, alewife, and zebrafish (Table 1). The relative molecular mass of thiaminase I extracted from alewife tissues and *P. thiaminolyticus* culture supernatant was determined by denaturing sodium dodecyl sulfate polyacrylamide gel electrophoresis (SDS-PAGE) followed by the thiaminase I activity assay (Supplementary Fig. S4). We were unable to estimate the denatured molecular mass of thiaminase I from common carp because it could not be renatured to an active form after electrophoresis. Using blue-native polyacrylamide gel electrophoresis (PAGE), we estimated the native size of the holoenzymes from *P. thiaminolyticus*, common carp tissues, and recombinant zebrafish candidate thiaminase I protein (Table 1, Supplementary Fig. S5). The recombinant zebrafish candidate thiaminase I protein exhibited bands from 25 to 100 kDa relative molecular mass, suggesting that the native holoenzyme is an oligomer composed of multiple subunits. In-gel native isoelectric focusing (IEF) followed by activity staining revealed differences in pI for all thiaminases investigated, and multiple isoforms in some species (Table 1, Supplementary Fig. S6). Co-substrate utilization assays showed that thiaminase I from common carp and alewife had no activity with pyridoxine as a co-substrate but had activity with both nicotinic acid and pyridine (Table 1, Supplementary Figs. S7 and S8). For all three fish tested, pyridine resulted in greater thiamine-degrading activity than nicotinic acid, in agreement with a previous study of common carp thiaminase I^28^. Thiaminase I from *P. thiaminolyticus* was active with all three co-substrates investigated.

**Table 1:** Summary of measured biochemical characteristics of thiaminases from this study, *Paenibacillus thiaminolyticus* (*Pt*, WP_087440168), common carp (*Cyprinus carpio*), alewife (*Alosa pseudoharengus*), and recombinant zebrafish (*Danio rerio*) putative thiaminase I protein (NP_001314821.1), and the literature, red cornetfish (*Fistularia* *petimba*)^27^. ^a^ND; not determined, ^b^By non-denaturing sodium dodecyl sulfate polyacrylamide gel electrophoresis (SDS-PAGE), ^c^By gel filtration, ^d^Only when not treated with heat.

	*Pt*	Common carp	Alewife	Recombinant zebrafish	Red cornetfish^27^
Molecular mass (M_r_, kDa)	42	ND^a^	23–25	24^b^	22 to 25
Relative molecular mass (M_r_, kDa) in native conformation	42	250	ND	25 to 100	106^c^
In-gel renaturation	Yes	No	Yes	Yes^d^	Yes
Number of isoforms (IEF)	3	4	1	2	2
Isoelectric point(s) (pI)	4.6; 9; 10	4.8; 5.7; 6.4; 9	5.0	3–6; 10	5.7; 7–9
Pyridoxine co-substrate activity	Yes	No	No	ND	No
Nicotinic acid co-substrate activity	Yes	Yes	Yes	Yes	No
Pyridine co-substrate activity	Yes	Yes	Yes	Yes	Yes

Empirically measured biochemical properties from thiaminase I extracted from alewife tissue matched predictions from the candidate gene sequence (Table 2). The calculated molecular masses and isoelectric points from the candidate thiaminase I protein sequences from common carp and alewife were in close agreement with measurements from thiaminase I activity in protein extracts from common carp and alewife tissues. The molecular weights observed are also in agreement with the molecular weight reported for thiaminase I activity in red cornetfish (Table 1)^27^.

**Table 2:** Comparison of measured biochemical characteristics of thiaminases with predictions from candidate gene sequences from common carp (*Cyprinus carpio*, XP_042594753), alewife (*Alosa pseudoharengus*, AleC120, Supplementary Fig. 2), and zebrafish (*Danio rerio*, NP_001314821). ^a^By non-denaturing sodium dodecyl sulfate polyacrylamide gel electrophoresis (SDS-PAGE).

	Common carp		Alewife		Recombinant zebrafish	
	Measured	Predicted	Measured	Predicted	Measured	Predicted
Molecular mass (M_r_, kDa)	ND	27.1	23–25	24.6	24^a^	26.1
Isoelectric point (pI)	4.8; 5.7; 6.4; 9	8.9	5.0	5.0	3–6; 10	8.9

## Discussion

We report here the discovery of a thiaminase I enzyme encoded in the genome of a multicellular animal and have demonstrated its thiaminolytic function in a recombinant system. We demonstrated that wild-type zebrafish tissues contain thiaminase I activity at levels similar to those previously documented in high-thiaminase I prey fish from Lake Michigan^16^. The thiaminase I of zebrafish is homologous to a candidate gene identified in alewife, and the empirically derived mass and isoelectric point of the thiaminase I activity extracted from alewife tissues exactly matched that predicted for the candidate alewife thiaminase I gene, demonstrating convergence across our two parallel approaches. This presents a compelling case for de novo synthesis of thiaminase I by alewife, a primary prey item for lake trout and other salmonine predators in the Great Lakes.

Our findings further confirmed previous reports that *P. thiaminolyticus* is not the source of thiaminase I activity in alewife^23^. Size estimates from denaturing SDS-PAGE revealed that thiaminase I extracted from alewife tissues was smaller than the *P. thiaminolyticus* thiaminase I and that the wild-type alewife thiaminase I is of the same size as that predicted from the alewife candidate thiaminase I gene and reported for red cornetfish thiaminase I^27^. Because the entire *P. thiaminolyticus* thiaminase I gene sequence is known^32^ and the structure has been crystalized^34^, there is no question that the 42 kDa mass of the *P. thiaminolyticus* protein represents the holoenzyme. Our results support the conclusion that the characterized zebrafish TenA-like thiaminase I originates in the zebrafish genome and is not a bacterial gene. The zebrafish TenA-like thiaminase I paralogs are included in the well-curated zebrafish reference genome, have intron–exon structure, and their expression as mRNA is supported by the zebrafish RefSeq RNA database. The homology of zebrafish TenA-like thiaminase I (NP_001314821.1) is much greater to other fish protein sequences than to bacterial TenA sequences. The biochemical characteristics of fish thiaminase I enzymes are similar to each other and different from bacterial thiaminase I enzymes. The enzyme activity of the fish thiaminase I enzymes are different from bacterial TenA proteins, which have thiaminase II activity.

Each thiaminase I we investigated had a unique pI. Common carp appears to have four isoforms that vary in pI across a rather wide range. Given the presumed oligomerization seen with the recombinant zebrafish thiaminase I, it is possible that one or more of the common carp isoforms were oligomers of the same monomer. The alewife thiaminase I focused in a very narrow range on IEF and therefore appears to consist of only one isoform. We included *P. thiaminolyticus* as a positive control for native IEF, and were surprised to find two heretofore unreported basic isoforms of *P. thiaminolyticus* thiaminase I. To our knowledge, multiple isoforms of thiaminase I in *P. thiaminolyticus* have not been demonstrated before. Interestingly, the recombinant zebrafish candidate thiaminase I was predicted to have pI of 8.9, and yet the majority of the activity is clearly in the acidic range. Prediction algorithms for pI’s are not as refined as those for mass, especially for basic proteins. Additionally, the use of the native IEF can result in an observed pI that differs relative to those obtained when using denaturing IEF with urea because unfolding of the protein can expose ionizable groups^35^.

The common carp and alewife candidate thiaminase I genes we identified did not produce soluble proteins in the recombinant *E. coli* system, and thus could not be tested for thiaminase I activity. Improper post-translational processing of eukaryotic proteins by prokaryotic expression systems is not uncommon. We have only indirect evidence that thiamine deficiency complex (TDC) resulting in early life stage mortality in lake trout in the Great Lakes is caused by maternal consumption of alewife expressing the TenA-like thiaminase I gene identified in the present study. However, we expect that the protein products of the candidate thiaminase I genes in alewife and common carp do encode thiaminase I enzymes for two reasons. First, the protein sequence alignments showed close relations with the zebrafish gene that we confirmed to produce thiaminase I activity. Second, the candidate thiaminase I gene for alewife is expected to produce a protein whose size and pI are exact matches to those we observed empirically in wild-type fish. Homologs to the zebrafish tenA-like thiaminase I are also found in other organisms known to have high thiaminase I activity, including Baltic herring (*Clupea harengus*, XP_031438567.1)^36^, American shad (*Alosa sapidissima*, XP_041935027.1)^37^, and zebra mussel (*Dreissena polymorpha*, KAH3819073.1)^38^ (Supplementary Tables S2 and S3).

Taken together, our biochemical and genetic findings suggest that each of the organisms we have investigated encodes a thiaminase I enzyme de novo. Our results do not exclude potential contributions to total thiaminase I activity in fish from intestinal microflora^39,40^ or dietary intake of thiaminase-rich prey. The in vivo expression and function of the zebrafish thiaminase I proteins have yet to be determined. The physiological function of thiaminase I is “a long-standing unsolved problem in thiamin physiology”^41^. Hypotheses include a role in the innate immune system to slow bacterial growth, protection against toxic thiamine analogs, an ecological function to limit predation, production of thiamine precursors for thiamine-synthesizing symbionts, or, if physiological conditions favor the reverse direction of the reaction, thiamine salvage. The presence of homologous genes across bacteria, fish, and mollusks suggests an ancient function that has been preserved in genomes across the tree of life. The function of thiaminase I may be more easily unraveled with modern genetic and proteomic tools now that the gene encoding the protein is known and a recombinant version of the protein is available. Knowing that fish are capable of producing thiaminase I de novo should inform future investigations for questions of fishery management importance such as understanding the factors that cause thiaminase I activity in fish to vary. Moreover, this information will help inform and guide human and wildlife health investigations of thiamine deficiency caused by thiaminase I-containing diets.

## Methods

### Sequence analysis

Protein sequence searches were conducted in the GenBank nr database with BLASTP^42^ using default parameters, including automatically adjusting parameters for short input sequences (Table S1). Conserved domain searches were run against the GenBank Conserved Domain Database (CDD)^43^. Sequence alignments were conducted in CLC Main Workbench 20.0.4 (Qiagen) with the fast alignment algorithm, gap open cost = 10, and gap extension cost = 1. Biochemical properties of the fish putative thiaminase I protein sequences were predicted with the Create Sequence Statistics function in CLC Main Workbench 20.0.4 (Qiagen, Hilden, Germany). The molecular weights were calculated from the sum of the amino acids in the sequence, and the isoelectric points (pIs) were calculated from the pKa values for the individual amino acids in the sequence.

### Bacteria culture

Pure cultures of *P. thiaminolyticus* strain 8188^22^ were cultured at 37 °C in Terrific Broth (MO BIO Laboratories, Carlsbad, CA) in either a shaking incubator or in a beveled flask with a stir bar and were harvested after 48–80 h of culture. Upon harvest, cultures were processed immediately or frozen whole in 50 mL Falcon tubes at − 80 °C. Fresh or thawed cultures were spun at 14,000×*g*, and culture supernatant was concentrated using Amicon-ultra 10 kDa molecular weight cut-off (MWCO) filters (EMD Millipore, Billerica, MA).

The zebrafish and alewife candidate thiaminase I genes were cloned and overexpressed in *E. coli* to determine whether they produced functional thiaminases. The recombinant thiaminase I gene from *P. thiaminolyticus* was overexpressed in *E. coli* as a positive control. Candidate and control genes were synthesized (Integrated DNA Technologies, Inc., Coralville, Iowa) and placed into the pET52b vector (EMD Millipore). Insert sequences are provided in Supplementary Figs. S10–S13. The empty pET52b vector was used as a negative control. The plasmid was transformed into *E. coli* (Rosetta 2(DE3)pLysS Singles Competent Cells, EMD Millipore) according to the manufacturer’s instructions, and expression of candidate genes was induced by the addition of IPTG. Cells were lysed in 1X BugBuster (Millipore) according to the manufacturer’s instructions in the presence of benzonase nuclease, and soluble and insoluble fractions were separated by centrifugation.

### Tissue collections

Adult common carp were captured from Lake Erie using short-set gill nets. Adult alewife and quagga mussels (*Dreissena bugensis*) were collected from Sturgeon Bay, Lake Michigan using bottom trawls. Fish collections were completed during July 2007. Sex of sampled fish was not identified. Upon collection, unanesthetized animals were immediately euthanized by flash freezing between slabs of dry ice and stored at − 80 °C. Fish were harvested by the Great Lakes Science Center, U.S. Geological Survey (USGS). Laboratory use of frozen animal tissues and wild type and recombinant bacteria was in accordance with institutional guidelines and biosafety procedures at Oregon State University and USGS. Animal care and use procedures were approved by the Great Lakes Science Center, USGS. All USGS sampling and handling of fish during research are carried out in accordance with guidelines for the care and use of fishes by the American Fisheries Society^44^. All methods are reported in accordance with applicable ARRIVE guidelines (https://arriveguidelines.org). Zebrafish from OSU's zebrafish facility were anesthetized and euthanized by overdose with waterborne 200 ppm ethyl 3-aminobenzoate methanesulfonate (MS-222, Sigma-Aldrich, St. Louis, MO) following protocols approved by the OSU Animal Institutional Care and Use Committee and were frozen at − 80 °C after euthanization. Gills, liver, spleen, and the intestinal tract were dissected, and gill tissue was homogenized separately from liver, spleen, and gut, which were homogenized together and designated “viscera.” Homogenization and protein preparation procedures were the same as that for alewife. Zebrafish from Columbia Environmental Research Center (CERC), USGS cultures were anesthetized and euthanized by overdose with 200 ppm ethyl 3-aminobenzoate methanesulfonate (MS-222, Sigma-Aldrich, St. Louis, MO) in water following protocols approved by CERC Institutional Animal Care and Use Committee (IACUC). Whole fish (0.2–0.6 g) were homogenized in 10 mL cold phosphate buffer, pH 6.5. Whole common carp and alewife were thawed until they could just be dissected. Preliminary trial extractions on alewife stomach and intestines, spleen, and gills revealed similar results and revealed that gills and spleen tissue produced the cleanest protein preparations. Therefore, subsequent extractions for common carp and alewife used gill tissue. Samples were pooled from 3 to 5 individual fish, haphazardly chosen from the sampled fish without exclusions. Quagga mussels were thawed just sufficiently to be husked from their shell and were used whole. Researchers were aware of the species and tissue designation of each sample throughout the experiments. Animal tissues were placed in ice-cold (4 °C) beakers containing cold extraction buffer (16 mM K_3_HPO_4_, 84 mM KH_2_PO_4_, 100 mM NaCl, pH 6.5 with 1 mM DTT, 2 mM EDTA, 3 mM Pepstatin, 1X Protease inhibitor cocktail (Sigma), and 1 mM AEBSF). All extractions were carried out at 4 °C in pre-chilled glassware. Samples were mechanically homogenized using a rotor–stator tissue grinder. Samples were stirred gently for several hours to overnight at 4 °C, centrifuged at 14,000×*g* to remove debris, and strained through cheesecloth to remove any insoluble lipids. Extracts were then subjected to 30–75% ammonium sulfate precipitation. Pellets from the precipitation were resuspended in buffer (83 mM KH_2_PO_4_, 17 mM K_2_HPO_4_, and 100 mM NaCl), centrifuged to remove any remaining debris, and stored in 30% glycerol at − 20 °C.

### Protein electrophoresis

Native PAGE was run using either pre-cast TGX gels (BioRad, Hercules, California) of varying percentage (7.5% to 12% or 8–16% gradient gels) or on hand-cast gels (TGX FastCast, BioRad) made according to the manufacturer’s instructions.

Blue-native PAGE was used to estimate the mass of thiaminases in their native conformation. Blue-native PAGE^45^ gels were run using the NativePage Novex Bis–Tris system (Life Technologies) or hand-cast equivalents^46^. Light blue cathode buffer was used to facilitate visualization of the activity stain.

Standard denaturing SDS-PAGE was used to estimate the molecular mass of thiaminases after denaturation. Denaturing SDS-PAGE was run using one of three relatively equivalent methods: pre-cast TGX gels (BioRad) according to the manufacturer’s instructions, hand-cast Tris–HCl gels using standard Laemmli chemistry^47^ with an operating pH of approximately 9.5, or hand-cast Bis–Tris gels (MOPS buffer) with an operating pH of approximately 7. For all denaturing and non-denaturing SDS-PAGE applications, standard Laemmli sample buffer was used, and samples were heated to 75 °C for 15 min to facilitate denaturation followed by brief centrifugation to eliminate any precipitated debris.

Non-denaturing PAGE was used as an alternative to denaturing PAGE for the common carp thiaminase that could not be renatured (i.e., activity could not be recovered) following a denaturing SDS-PAGE. Non-denaturing PAGE was conducted using any of the three aforementioned gel chemistries with SDS-containing running buffers including reductant (DTT), but samples were not heated prior to application to the gel. Samples for non-denaturing PAGE were allowed to incubate in sample buffer at room temperature for 30 min prior to gel loading. This preserves the charge-shift induced by SDS but does not result in protein denaturation, facilitating in-gel analysis of thiaminase I activity after separation.

To visualize proteins following electrophoresis, gels were stained with Coomassie stain (CBR-250 at 1 g/L in methanol/acetic acid/water (4:5:1) and destained with methanol/acetic acid/water (1.7:1:11.5). Mini-gels were run on BioRad’s mini-protean gel rigs. Midi-gels (16 cm length) were run on Hoefer’s SE660, and large-format gels (32 cm length) were run on a BioRad’s Protean Slab Cell. Mini-gels were generally run at room temperature, and midi- and large-format gels were run at 4 °C. Blue-native PAGE was always run at 4 °C.

Two-dimensional electrophoresis (2DE) separated proteins in the first dimension based on pI and in the second dimension based on mass (either native or denatured). 2DE was performed by combining in-gel IEF with either denaturing SDS-PAGE, non-denaturing SDS-PAGE, or native PAGE. IPG strips were incubated in TRIS-buffered equilibration solution^48^ either with 6 M urea, SDS, and iodacetamide (denaturing) or without urea, SDS, and iodacetamide (non-denaturing) for 20 min. Low melting point agarose was used to solidify IGP strips in place. Agarose was cooled to just above the gelling temperature, as hot agarose inactivated thiaminase I activity.

### Isoelectric focusing

Isoelectric focusing (IEF) was conducted both in-gel and in-liquid. In-gel IEF was conducted in immobilized pH gradient (IPG) strips using a Multifor II (GE Healthcare Life Sciences). Prior to rehydration, all protein preparations were desalted in low-salt (~ 5 to 10 mM) sodium or potassium phosphate buffer (pH 6.5) using 10 kDA MWCO filters. All samples were applied using sample volumes and protein concentrations recommended by the manufacturer. For standard denaturing in-gel IEF, rehydration solution consisted of 8 M urea, 2% CHAPS, 2% IPG buffer of the appropriate pH-range, 1% bromophenol blue, and 18 mM DTT. The IEF was conducted at maximum of 2 mA total current and 5 W total power, with an EPS3500 XL power supply in gradient mode. Voltage gradients were based on standard protocols recommended by the manufacturer. In-gel IEF was also performed under native conditions to allow thiaminase I activity staining of IPG strips. Protocols were essentially the same as those for denaturing conditions, with the following exceptions: (1) urea was eliminated and the CHAPS concentration was reduced to 0.5% in the rehydration solution; (2) rehydration was conducted at 14 °C; and (3) the water in the cooling tray was cooled to 4 °C.

In-liquid IEF was conducted using a Rotofor (BioRad) according to the manufacturer’s instructions. Non-denaturing in-liquid IEF was also conducted using a focusing solution including no urea, 2% pH 3–10 biolyte, 0.5% CHAPS, 20% glycerol, and 5 mM DTT. The addition of glycerol helped retain activity but also increased focusing times. The Rotofor was run at a constant 15 W with a maximum current of 20 mA and voltage set for a maximum of 2000 V. Samples containing 8 M urea were cooled to 14 °C during focusing to avoid urea precipitation, whereas samples lacking urea were cooled to 4 °C during focusing. Protein extracts in salt solutions greater than 10 mM were desalted directly in focusing solution using a 10 kDA MWCO filter. Focusing runs were allowed to proceed until the voltage stabilized and fractions were harvested with the needle array and vacuum pump. Ampholytes were removed by addition of NaCl to 1 M and then samples were desalted into phosphate buffer using a 10kD MWCO filter.

### Thiaminase I activity measurements

For quantitative measurements of thiaminase I activity, we conducted a radiometric assay at CERC as previously described^49^. Zebrafish homogenates were diluted 1:8, 1:16, or 1:32 in cold phosphate buffer, pH 6.5. Two replicates per dilution were assayed. Activity was calculated from the greatest dilution that gave activity within the linear range of the assay and was reported as pmol thiamine consumed per g tissue (wet weight) per minute (pmol/g/min).

### Thiaminase I activity staining

After electrophoresis, gels were stained for thiaminase I activity using a previously described diazo-coupling reaction^19,50^. Briefly, gels were washed 3 times in water, twice in 25 mM sodium phosphate buffer with 1 mM DTT, and once in 25 mM sodium phosphate buffer without DTT. Gels were then incubated in 0.89 mM thiamine-HCl and co-substrate (1.45 mM pyridoxine, 24 mM nicotinic acid, or 20 mM pyridine) in 25 mM sodium phosphate buffer for 10 min. Gels were briefly rinsed in water and placed in a lidded container and incubated at 37 °C for 30 min to allow thiamine degradation by any thiaminases in the gel. The diazo stain^19,50^ was then applied to detect remaining thiamine in the gel for five minutes with gentle agitation. Stained gels were rinsed with water and photographed, and further stained with Coomassie to visualize proteins.

## Supplementary Information

Supplementary Information.

## Data Availability

The datasets generated and/or analysed during the current study are publicly available from the U.S. Geological Survey repository, https://doi.org/10.5066/P9DGI5F5^29,33^.
