# Supplementary material for: Genetic basis of thiaminase I activity in a vertebrate, zebrafish Danio rerio
Source: Sci Rep. 2023 Jan 13;13:698. doi: 10.1038/s41598-023-27612-5 (PMC9839694; doi:10.1038/s41598-023-27612-5)
Supplement: Supplementary file 1 — Supplementary Information. [file 41598_2023_27612_MOESM1_ESM.docx]

**Supplementary Information for**

**Genetic basis of thiaminase I activity in a vertebrate, zebrafish *Danio rerio***

**Catherine A. Richter^1,†,^*, Allison N. Evans^2,3,†^, Scott A. Heppell^2^, James L. Zajicek^1^, and Donald E. Tillitt^1^**

^1^U.S. Geological Survey, Columbia Environmental Research Center, Columbia, Missouri 65201, USA

^2^Department of Fisheries, Wildlife, and Conservation Sciences, Oregon State University, Corvallis, Oregon 97331, USA

†Authors contributed equally

^3^Current affiliation: Department of Microbiology, Oregon State University, Corvallis, Oregon 97331, USA

* [CRichter@usgs.gov](mailto:CRichter@usgs.gov)

Any use of trade, firm, or product names is for descriptive purposes only and does not imply endorsement by the U.S. Government.

**
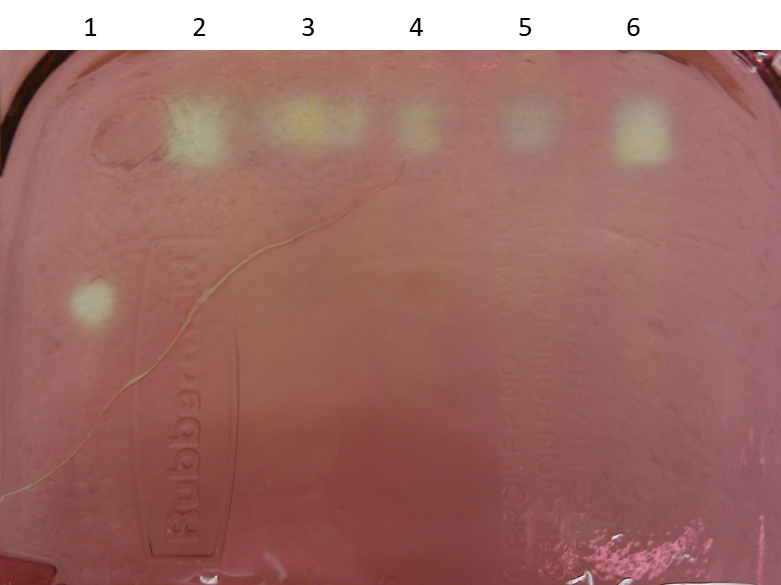
**

**Supplementary Figure S1.** Native polyacrylamide gel electrophoresis (PAGE)showing holoenzyme mobility of thiaminase I from different species and tissue preparations. Activity stain with pyrimidine co-substrate shows thiamine degradation as clear areas. Lane 1, *Paenibacillus thiaminolyticus* culture supernatant; Lane 2, common carp (*Cyprinus carpio*) homogenate; Lane 3, zebrafish (*Danio rerio*) viscera (40 ug); Lane 4, zebrafish viscera (20 ug); Lane 5, zebrafish viscera (10 ug); Lane 6, zebrafish gill (20 ug). Photo Credit: Allison Evans, Oregon State University.

**A**

>AleC120 from alewife 454 cDNA [organism=Alosa pseudoharengus]

TTCATGTGAAGAAGCTTTCGTGGTGTGTTAACTGGACTGCACTTCTGAGTGATTCAGTGACAGTGGCAATACAGACAGAAAATGCAGTCAGAAAACAAAGATGTGTATGACCTCCTCTGGGACAACAATATGGATATTGCTGAGCAGACATTGGAAGTGCCATTTCTACAGCACATGCAGCTTGGAGACCTCCAGGCTGACGACTATGTGAGCTTCACCATCCAGGACATCAATTACCTTGTGAGGGTAACAGACATGTTGGGGGAGATGTGTGAGAAAGGAAAGCTACCAGAGGATCTCCACGGGTTCATGAAGGAGAGATATGACAGCTATAACAACTACGCTGTTGCTACACTACAGCAATTTAACCTAAATAGTGTGTCGGATATTAAACCAACCCCTGCCATGGAGAAGTACCTGTCAAATTACAGCGATATCATGGAGGGAGAAGAAGCCATTTACTTTGCAGTCGCTCTCCTTCCCTGCTCAGTGCTGTGGCTGTGGCTGGCCAAACAGCTGAAAGAAACCACTTGCAACGCGTACTTCACCTGGAAGAAGAACAACATGCACGGCCACCCAGAAGACCACTACAGGGCCCTCCTCGACAAGTATCTGACCACTAAAGAGCAGATTGCAAAGGCTGACACCATATTCCGTCAACAAATGCAGAATGAGCATGATTTCTTTGCATCTTCACTCATAGAGAAGAAATAAAATGACAGTGTACTGTATGTCACCTGTCACACCATCAAGTTTAAGTTCAAGTAATTTGTCACACATATAGTAGGCCTATGTAATAACAGTGAAATAAAGGTTGTAGCCACTCCTGACTGTGCAACAGAAAG

**B**

> AleC120 from alewife 454 cDNA [organism=Alosa pseudoharengus] translation_frame_+1

MQSENKDVYDLLWDNNMDIAEQTLEVPFLQHMQLGDLQADDYVSFTIQDINYLVRVTDMLGEMCEKGKLPEDLHGFMKERYDSYNNYAVATLQQFNLNSVSDIKPTPAMEKYLSNYSDIMEGEEAIYFAVALLPCSVLWLWLAKQLKETTCNAYFTWKKNNMHGHPEDHYRALLDKYLTTKEQIAKADTIFRQQMQNEHDFFASSLIEKK

**Supplementary Figure S2.** Expressed nucleotide sequence (A) and predicted protein sequence (B) of putative alewife (*Alosa pseudoharengus*) tenA-like thiaminase I. Sequence from S. Czesny, J. Epifanio, P. Michalak, Genetic divergence between freshwater and marine morphs of alewife (*Alosa pseudoharengus*): A ‘next-generation’ sequencing analysis. *PLOS ONE* 7, e31803 (2012).


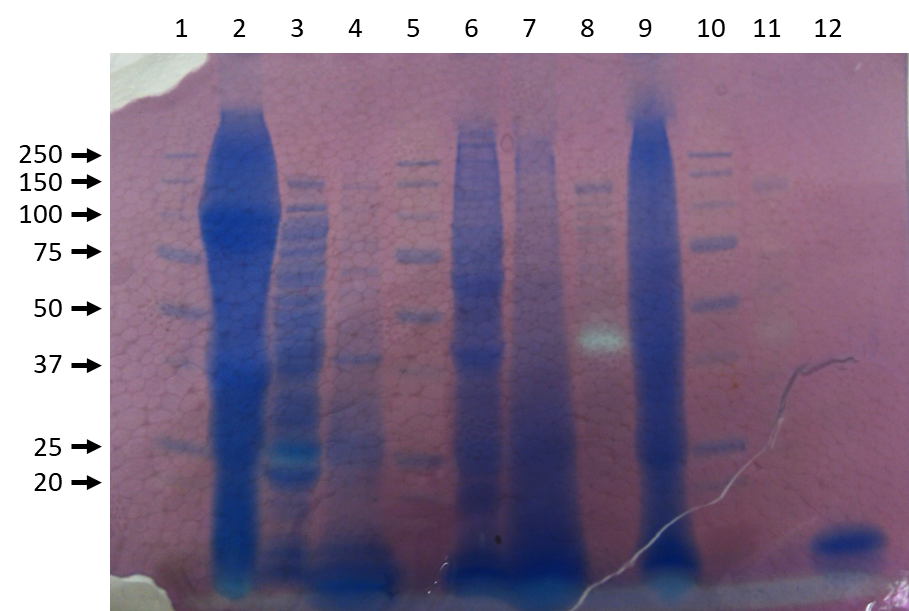


**Supplementary Figure S3.** Sodium dodecyl sulfate polyacrylamide gel electrophoresis (SDS-PAGE) without heat denaturation of different protein preparations. Thiaminase I activity stain with pyrimidine co-substrate shows thiamine degradation as clear areas. Lanes 1, 5, and 10, Molecular weight standards (20, 25, 37, 50, 75, 100, 150, 250 kDa); Lane 2, zebrafish (*Danio rerio*) viscera; Lane 3, recombinant zebrafish candidate thiaminase I protein sequence NP_001314821.1 overexpressed in *Escherichia coli*; Lane 4, common carp (*Cyprinus carpio*) homogenate fractionated for pI >9; Lane 6, common carp homogenate; Lane 7, quagga mussel (*Dreissena bugensis*) homogenate, Lane 8, *Paenibacillus thiaminolyticus* culture supernatant; Lane 9, alewife (*Alosa pseudoharengus*) homogenate; Lane 11, *P. thiaminolyticus* culture supernatant fractionated for pI 4-6. Photo Credit: Allison Evans, Oregon State University.


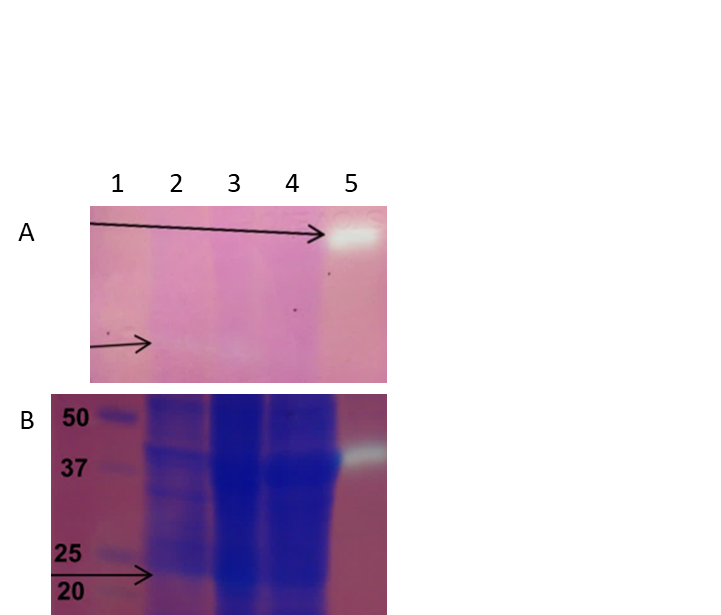


**Supplementary Figure S4.** Relative molecular weight determination of thiaminases from alewife (*Alosa pseudoharengus*) and *Paenibacillus thiaminolyticus*, separated by denaturing polyacrylamide gel electrophoresis (PAGE). A) Activity stain showing thiamine degradation as clear areas (arrows). B) Activity stain with additional Coomassie blue protein stain of the same gel as panel A. Lane 1, molecular weight markers; 2, alewife spleen; 3, alewife gill; 4, alewife gut; 5, *P. thiaminolyticus* culture supernatant. Photo Credit: Allison Evans, Oregon State University.


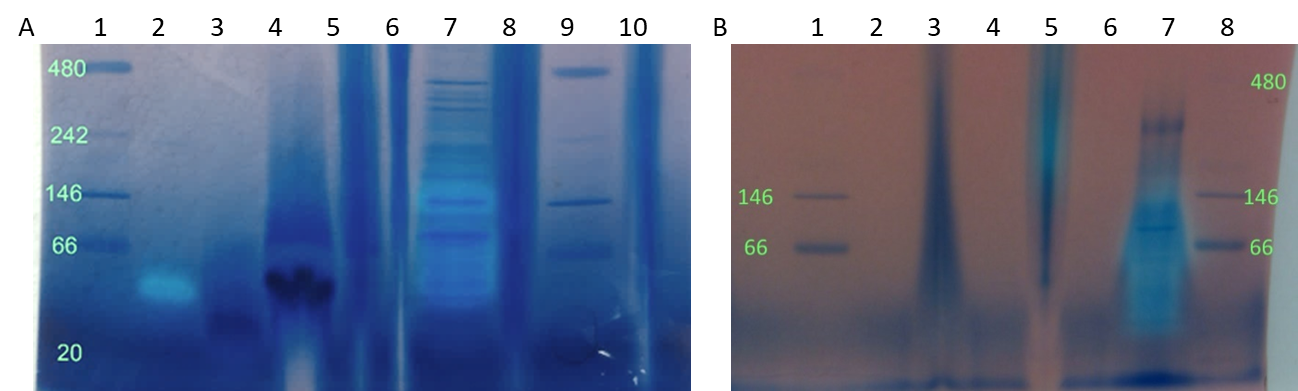


**Supplementary Figure S5.** Relative holoenzyme size assessment by blue-native polyacrylamide gel electrophoresis (PAGE) of thiaminase I from *Paenibacillus thiaminolyticus*, recombinant candidate thiaminase I protein from zebrafish (*Danio rerio*), and thiaminase I from common carp (*Cyprinus carpio*) tissue protein extract; stained for activity showing thiamine degradation as clear areas, with additional Coomassie blue protein stain. Pyridine was used as the co-substrate for the thiaminase activity assay. Panel A: Lane 1, molecular weight markers; Lane 2, *P. thiaminolyticus* culture supernatant; Lane 7, recombinant zebrafish putative thiaminase protein. Panel B: Lane 1, molecular weight markers; Lane 5, common carp tissue protein extract; Lane 7, recombinant zebrafish putative thiaminase protein; Lane 8 molecular weight markers. Unlabeled lanes are not relevant to the results but are shown to avoid distorting the gel image. The gel in panel A used dark blue cathode buffer, and the gel in panel B used light blue cathode buffer. Photo Credit: Allison Evans, Oregon State University.


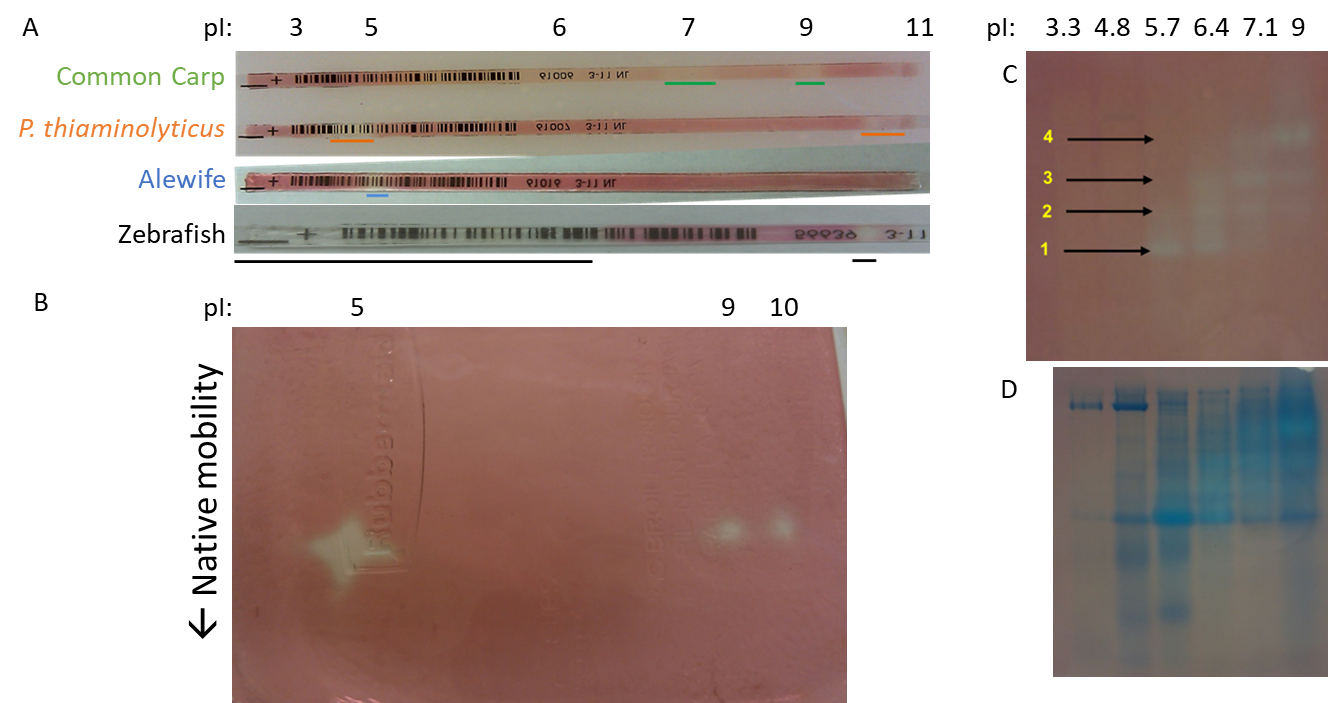


**Supplementary Figure S6.** Native isoelectric point comparison among thiaminases. White areas on the red background indicate thiaminase activity. Numbers at the top indicate approximate pI. A) Thiaminase I from each sample focused on a separate IPG strip. B) Native 2-dimensional gel of *Paenibacillus thiaminolyticus* culture supernatant. C) Native gel of separate liquid IEF fractions of common carp (*Cyprinus carpio*) protein extract, with 4 isoforms (arrows). Labels at the top of the gel indicate the average pH of the liquid fractions from in-liquid IEF. D) Additional Coomassie blue protein stain of the same gel as panel C. Photo Credit: Allison Evans, Oregon State University.


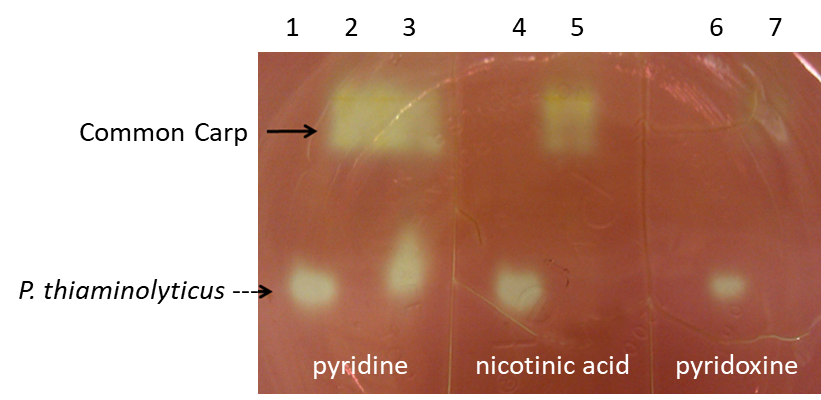


**Supplementary Figure S7.** Thiaminase I from common carp (*Cyprinus carpio*) and *Paenibacillus thiaminolyticus* protein extracts show differential co-substrate utilization patterns. Image is of a single gel cut into three segments and separately activity stained with different co-substrates. Lanes 1-3 were assayed with pyridine as the co-substrate, lanes 4-5 were assayed with nicotinic acid as the co-substrate, and lanes 6-7 were assayed with pyridoxine as the co-substrate. Activity stain shows thiamine degradation as clear areas. Lanes 1, 4, 6, *P. thiaminolyticus*; lanes 2, 5, 7, common carp, Lane 3 mixture of *P. thiaminolyticus* and common carp. Photo Credit: Allison Evans, Oregon State University.


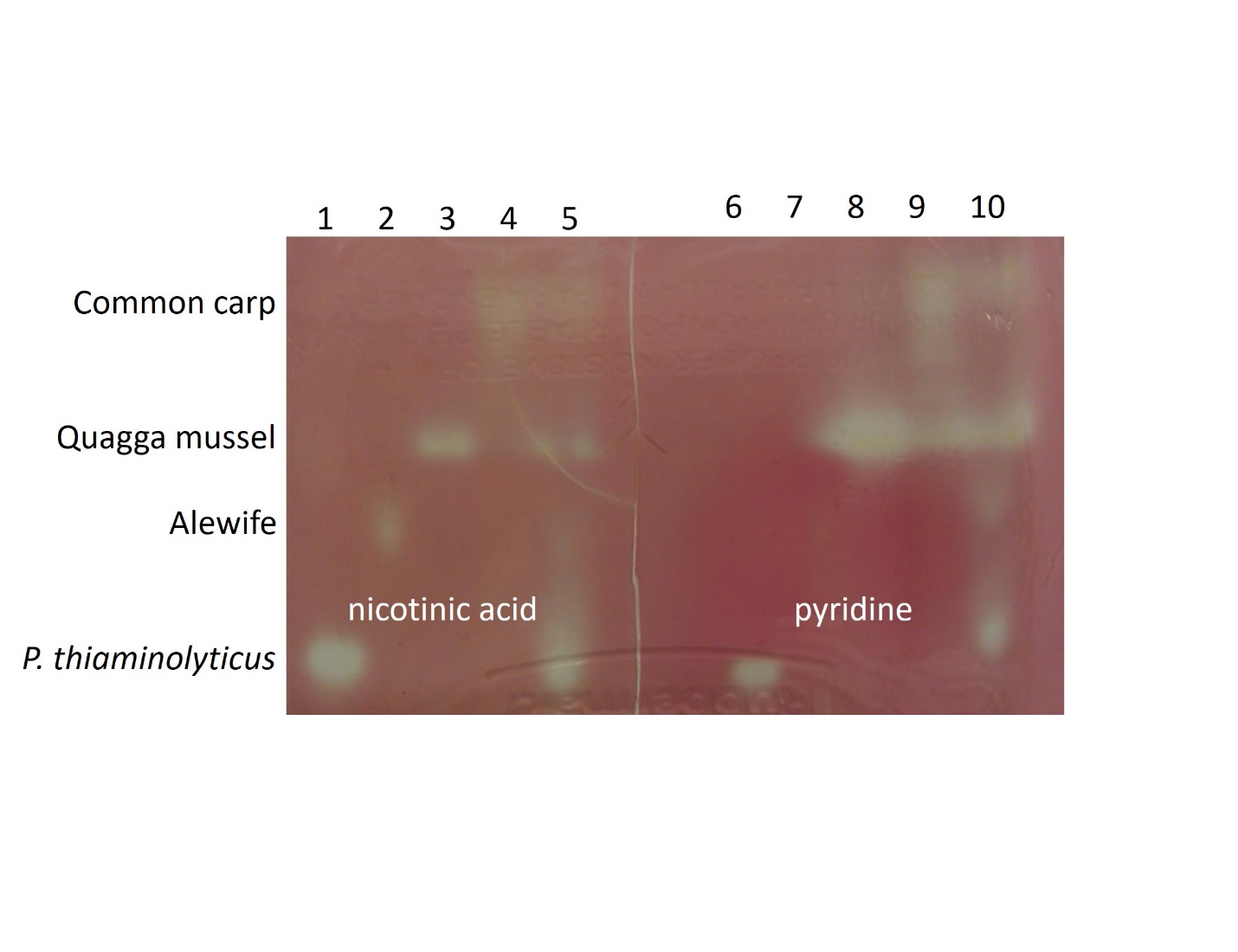


**Supplementary Figure S8.** Co-substrate utilization patterns of thiaminase I from different sources. Image is of a single gel cut into two segments and separately activity stained with different co-substrates. Lanes 1-5 were assayed with nicotinic acid as the co-substrate, lanes 6-10 were assayed with pyridine as the co-substrate. Activity stain shows thiamine degradation as clear areas. Lanes 1, 6, *Paenibacillus thiaminolyticus* culture supernatant; lanes 2, 7, alewife (*Alosa pseudoharengus*), band in lane 7 is very faint because of a loading error; lanes 3, 8, quagga mussel (*Dreissena bugensis*); lanes 4, 9, common carp (*Cyprinus carpio*); lanes 5, 10, mixture of *P. thiaminolyticus* culture supernatant, alewife, quagga mussel, and common carp. Photo Credit: Allison Evans, Oregon State University.

**A**


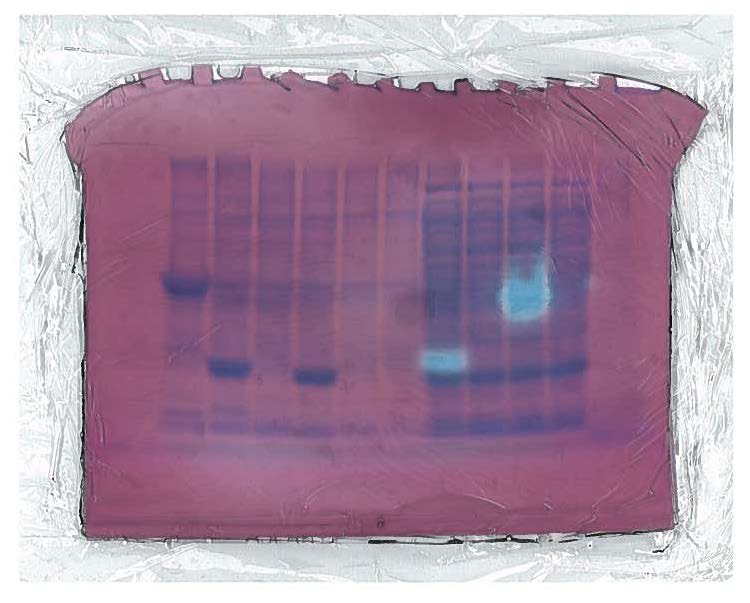


5

4

3

2

1

**B**


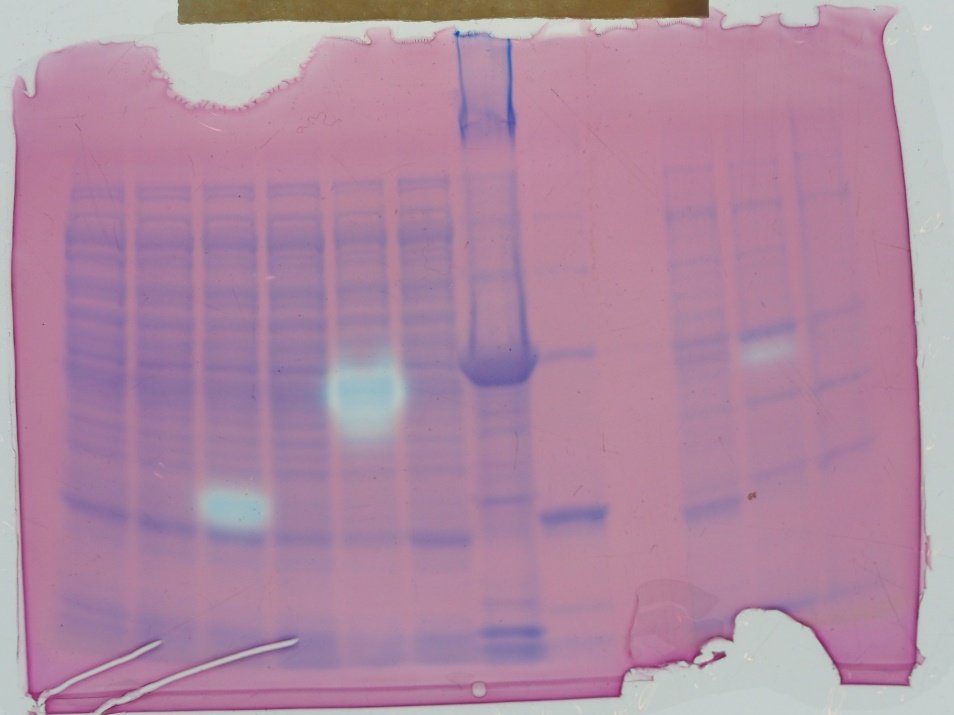


5

4

3

2

1

**Supplementary Figure S9.** **The zebrafish (*Danio rerio*) tenA-like thiaminase I gene encodes a functional thiaminase.** Solubility and thiaminase I activity of recombinant candidate thiaminase I genes overexpressed in *Escherichia coli*, stained for activity showing thiamine degradation as clear areas, and with subsequent Coomassie blue protein stain. Gels are cropped to show relevant lanes and mobility range; original gels are presented in Supplementary Figure 9. Panel A, insoluble fractions; Panel B, soluble fractions; lane 1, common carp (*Cyprinus* *carpio)*, lane 2, zebrafish; lane 3, alewife (*Alosa pseudoharengus*); lane 4, *Paenibacillus thiaminolyticus*; lane 5, empty pET52b vector.

**A**

> zebrafish TenA-like thiaminase I insert DNA sequence

CCATGGAAGACGTCTACGAATACTTGTGGCAAAAGAACAAGGATTTGGCTGTTCAAACCTTGAACTTGGATTTCTTGAGACAAATGGAATCTGGTTCCTTGCAAGCTGAAAGATACGTTAACTTCACCATCCAAGATATCGGTTACGTTTTGGCTGTTACCAAGATGTTGAAAAGAATGTCTGCTGAAGTGTCCCAACCAGATGATATTAGAGATTTCATGAAGGGTAGATTCGCTTCTTACAAGTCTTTCGGTGAGTTGTTGTTGAATATGTACTTCTTCAAGGCTGAACCACCAATCCAAAGAACTCCAGCTTTGAGAAATTACTTGTTGTCTTACAGATTCTTGATGTTCGAAGAACCTATCTACTTCGTTGTTGGTTTGTTGCCATGTGCTAGATTGTGGGTTTGGTTGGCTAACAATTTGAACATCCCACAAACTAACGCTTACTTCACTTGGAAGGTTGATAATATGGGTGGTCATCCAGAAAAACATTACAAGGCTTTGTTGAACAAGTACTTGAACACCGCTGATAAGGTTGCTAAAGCTAATGCTGTTTTCAGAGATCAAATGCAAAACGAGTACAAGTTCTTCTTGACCTTCGAGCTC

**B**

> zebrafish TenA-like thiaminase I insert translation_frame_+3

MEDVYEYLWQKNKDLAVQTLNLDFLRQMESGSLQAERYVNFTIQDIGYVLAVTKMLKRMSAEVSQPDDIRDFMKGRFASYKSFGELLLNMYFFKAEPPIQRTPALRNYLLSYRFLMFEEPIYFVVGLLPCARLWVWLANNLNIPQTNAYFTWKVDNMGGHPEKHYKALLNKYLNTADKVAKANAVFRDQMQNEYKFFLTFEL

**Supplementary Figure S10.** Insert sequence cloned into pET52b+ vector for expression of the putative zebrafish (*Danio rerio*) TenA-like thiaminase I protein sequence (NP_001314821.1).

**A**

> common carp TenA-like thiaminase I insert DNA sequence

CCATGGAAGACGTCTACGAATTCATTTGGGAAAACAACATCGATATCGCTTACAAGACTATCAGAGGTGATTTCTTGATCCAAATGCAAAACGGTTCATTGCAAGCTGAAAGATATATCTCCTTCACCATCCAAGATTTGAATTACGTTTTGAAGGTTGCTGAAATGTTGAAAAAGATGTCTGCTAACGTTACCCAACCTAACGATTTGAAGGATTTCTTGAACGGTAGATACTCCTCTTATAAGGGTTTCGGTAACTTGATGTTGAAGCAATACTTCTTCAAAGGTGAACCACCAATCGAACAAACTCCAGCTATGAAGAAATACTTGGCTTACTACAGAAACTTGATGGATAACGAAGAACCATTATACTTCGCTGTTGGTTTGTTGCCATGTGCTAGATTGTGGGTTTGGTTGGCTAAGAATTTGAACACTCCATCAACTAACGCTTACTACACTTGGAAGGTTGAAAACATGGGTGGTCATCCAGAAAAACATTACAGAGCTTTGTTGAACAAGTACTTGAACACTACCAAGACCGTTGAAAAGGCTAACGCTATTTTCAGAGCACAAATGCCAAACGAACATGATTTCTTTTTGTCCTCTGAGCTC

**B**

> common carp TenA-like thiaminase I insert translation_frame_+3

MEDVYEFIWENNIDIAYKTIRGDFLIQMQNGSLQAERYISFTIQDLNYVLKVAEMLKKMSANVTQPNDLKDFLNGRYSSYKGFGNLMLKQYFFKGEPPIEQTPAMKKYLAYYRNLMDNEEPLYFAVGLLPCARLWVWLAKNLNTPSTNAYYTWKVENMGGHPEKHYRALLNKYLNTTKTVEKANAIFRAQMPNEHDFFLSSEL

**Supplementary Figure S11.** Insert sequence cloned into pET52b+ vector for expression of the putative common carp (*Cyprinus carpio*) TenA-like thiaminase I protein sequence (XP_042594753).

**A**

> alewife TenA-like thiaminase I insert DNA sequence

CCATGGCACAATCCGAAAACAAGGATGTGTACGATTTGTTGTGGGATAACAACATGGATATCGCTGAACAAACTTTGGAAGTGCCATTCTTGCAACATATGCAATTGGGTGATTTGCAAGCTGATGATTACGTTTCTTTCACCATCCAAGATATCAACTACTTGGTTAGAGTGACCGATATGTTGGGTGAAATGTGTGAAAAAGGTAAATTGCCTGAAGATTTGCATGGTTTCATGAAGGAAAGATACGATTCCTACAACAACTACGCTGTTGCTACATTGCAACAATTCAACTTGAACTCCGTGTCTGATATTAAGCCAACTCCAGCTATGGAAAAGTACTTGTCTAACTACTCCGATATCATGGAAGGTGAAGAAGCTATCTATTTCGCTGTTGCTTTGTTGCCATGTTCTGTTTTGTGGTTGTGGTTGGCTAAGCAATTGAAAGAAACTACCTGTAACGCTTACTTCACCTGGAAAAAGAACAACATGCATGGTCATCCAGAAGATCATTACAGAGCTTTGTTGGATAAGTACTTGACCACCAAAGAACAAATCGCTAAGGCTGATACCATCTTCAGACAACAAATGCAAAACGAACATGATTTCTTCGCTTCATCTTTGATCGAAAAAAAGGAGCTC

**B**

> alewife TenA-like thiaminase I insert translation_frame_+3

MAQSENKDVYDLLWDNNMDIAEQTLEVPFLQHMQLGDLQADDYVSFTIQDINYLVRVTDMLGEMCEKGKLPEDLHGFMKERYDSYNNYAVATLQQFNLNSVSDIKPTPAMEKYLSNYSDIMEGEEAIYFAVALLPCSVLWLWLAKQLKETTCNAYFTWKKNNMHGHPEDHYRALLDKYLTTKEQIAKADTIFRQQMQNEHDFFASSLIEKKEL

**Supplementary Figure S12.** Insert sequence cloned into pET52b+ vector for expression of the putative alewife (*Alosa pseudoharengus*) TenA-like thiaminase I protein sequence (AleC120, Supplementary Figure S2).

**A**

> *P. thiaminolyticus* thiaminase I insert DNA sequence

TTCCTCTAGAATAATTTTGTTTAACTTTAAGAAGGAGATATACCATGTCAAAGATAAAAGGCTTCATTTATAAGCAATTCATGGTTATGCTGGTATTGCTGCTCGTGGTTGTCAGTCCGGCCGGAGCCGGGGCGGCTCTTTCCGACGCTTCTTCCGATATTACGTTGAAGGTGGCAATCTATCCGTACGTGCCCGATCCTTCCCGTTTCCAAGCAGCCGTCCTTGACCAGTGGCAGCAGCTAGAGCCTGGCGTCAGGCTGGAGTTTACGGAATGGGATTCCTATTCCGAGGATCCGCCGGATGATTTGGATGTGTTCGTGCTGGATTCTATTTTTTTAAGCCATTTTGTCGATGGGGGGTATCTGCTGCCCTTCGACAGCCAAGATATCGATCAGGCGGAGGATGTAATCCCTTTTGCTCTGCAAGGAGCGACGCGCAACGGCAAGGTATACGGCCTGCCGCAAATTTTGTGCACGAACCTGCTTTTTTACCGGAAAGGCGATTTGAAGATAGGGCAAGTCAACAATATATATGAGCTGTACAAAAAAATAGGAACCAACCATTCCGAGCAGATTCCGCCTTTGAAAAATAAGGGCTTGTTAATTAATATGGCTGGCGGGACGACGAAGGCGAGCATGTATTTGGAGGCGCTTATCGATGTGACTGGCCAGTATACGGAATATGACCTTCTTCCGCCGCTCGACCCCCTGAATGACAAAGTCATTCGCGGCTTGCGGCTGCTAATTGATATGGCGGGGGAGAAGCCGTCTCAGTATGCTCCTGAGGATGGCGACTCCTATGTAAGAGCGTCGTGGTTCGCGCAAGGCAGCGGAAGGGCCTTCATCGGCTACAGCGAGTCGATGATGCGCATGGGCGAGTATGCGGATCAGGTCCAGTTCAAGACGATTTCCTCATCGGCGGGGCAGGACATTCCTCTTTTCTACAGCGATGTCGTGAGCGTGAACTCCAAGACGGCCTATCCGGAGCTGGCCCAAAAACTGGCGAATATTATGGCTTCCGCGGATACGGTAGAGCAAGCTCTGCGCCCGCAGGCCGATGGCCAATATCCACAGTATCTGTTGCCTGCCCGGCATCAGGTATACGAAGCATTGATGCAAGATTATCCGATTTATTCCGAATTGTCGGGTATCGTGAACCAGCCGTCGAATCGTGTGTTCCGGCTTGGGCCTGAAGTGCGCACGTGGCTGAAGGATGCCAAGCGAGTGCTGCCCGAAGCGCTCGGCTTGACGGACGCCTCGAGCCTGGCGAGCGAGCTCGCTCTGGTGCCACGCGGTAGTTCCGCTCATCACCACCATCATCACCATCACCACCACTA

**B**

> *P. thiaminolyticus* thiaminase I insert translation_frame_+3

FCLTLRRRYTMSKIKGFIYKQFMVMLVLLLVVVSPAGAGAALSDASSDITLKVAIYPYVPDPSRFQAAVLDQWQQLEPGVRLEFTEWDSYSEDPPDDLDVFVLDSIFLSHFVDGGYLLPFDSQDIDQAEDVIPFALQGATRNGKVYGLPQILCTNLLFYRKGDLKIGQVNNIYELYKKIGTNHSEQIPPLKNKGLLINMAGGTTKASMYLEALIDVTGQYTEYDLLPPLDPLNDKVIRGLRLLIDMAGEKPSQYAPEDGDSYVRASWFAQGSGRAFIGYSESMMRMGEYADQVQFKTISSSAGQDIPLFYSDVVSVNSKTAYPELAQKLANIMASADTVEQALRPQADGQYPQYLLPARHQVYEALMQDYPIYSELSGIVNQPSNRVFRLGPEVRTWLKDAKRVLPEALGLTDASSLASELALVPRGSSAHHHHHHHHHH

**Supplementary Figure S13.** Insert sequence cloned into pET52b+ vector for expression of the *Paenibacillus thiaminolyticus* thiaminase I protein sequence (WP_087440168).

**A**

> common carp corticosteroid-binding globulin-like isoform X1 insert DNA sequence

TCGAAAAGGGTGCACTTGAAGTCCTCTTTCAGGGACCCGGGTACCAGGATAAGTTGCCATCTTTGATCAAGATGAACAACGATTTCGCTTTCCACTTGTACAAGAGATTGGTGGAAATGCCAGAATACCAATCCAAGAACATTTTCTTCTCTCCATTCTCTGTGTCCATGGCTTTGTCTGAATTGTCTTTGGGTGCTGGTGGTGAAACAAAAGAACAATTATTGTCTGGTATCGGTCATAACTCCTCTGTTTTCTCTACTGAAGAAATGCATCAAATGTTCCATTCTTTGTTGGAAGAAATCGATCAAAGAACCGGTGTGGATATTAACGTTGGTTCTGCATTATACGCTTCCGATAAGTTGAAGTTGTTGCCAGAATTCTTGAAAGAAATCAAAGAATTCTACCATTCTGATGGTTTCACCGTGGATTTCTCTGTGAAAGAAACTTTGGATAAGATCAACACCTACGTGAAAGAAAAGACCCATGGTAAGATTGATCAAGCTGTGGATGATTTGGAATCCGATACTTTGATGTTCTTGTTGACCTACATCTACTTCAAGGGAAAATGGGATATGCCATTCAACCCATCTAAGACCTCTCAATCTAGATTCCATGTTGATGCTGAAACTACCGTTCCAGTTCAAATGATGCATCAGTACAAGTCCTTGAAGGTGTACTACGATGTTGAATTGACCTCTAAGGTGTTGTGTTTGGATTACAACGATTCTTTCTCCATGTTCTTGGCTGTTCCAGATACTGATAGACCAGCTAAGACTATCAAGGATTTGGAAATGGCTATCTCTAGACAACATATCGAAAAGTGGAGATCTGCTGTGAGAAAGAGAAAGACTGATATCTTCGTTCCAAAGTTGTCCTTGAAAACCACCTACTCATTGAAGGATATCTTGAAGGGTATGGGTATGGCTGATATGTTCTCTTACAGAGCTAACTTCACTGGTATCTCCGAAGAAAACATGTTGATCTCAAAGGTGTTGCATAAGGCTTCATTGGATATCGACGAAAAGGGTACTACTGCTGCTGCTGTTACTACTGTTGATTTCAGACCAATGTCTTACTCTCCATTGGATACCTTGTCTTTCGATAGACCATTCATGATCTTCATCACCGATCAAAAGATGACCACCTCTTCATCTTTGGAAAAGTTGTCTATTAGAAGAAAGAACTCCAACGTGGCTTTCAAGATGTCTGAGCTCGCTCTGGTGCCACGCGGTAGTTCCGCTCATCACCACC

**B**

> common carp corticosteroid-binding globulin-like isoform X1 insert translation_frame_+3

EKGALEVLFQGPGYQDKLPSLIKMNNDFAFHLYKRLVEMPEYQSKNIFFSPFSVSMALSELSLGAGGETKEQLLSGIGHNSSVFSTEEMHQMFHSLLEEIDQRTGVDINVGSALYASDKLKLLPEFLKEIKEFYHSDGFTVDFSVKETLDKINTYVKEKTHGKIDQAVDDLESDTLMFLLTYIYFKGKWDMPFNPSKTSQSRFHVDAETTVPVQMMHQYKSLKVYYDVELTSKVLCLDYNDSFSMFLAVPDTDRPAKTIKDLEMAISRQHIEKWRSAVRKRKTDIFVPKLSLKTTYSLKDILKGMGMADMFSYRANFTGISEENMLISKVLHKASLDIDEKGTTAAAVTTVDFRPMSYSPLDTLSFDRPFMIFITDQKMTTSSSLEKLSIRRKNSNVAFKMSELALVPRGSSAHHH

**Supplementary Figure S14.** Insert sequence in pET52b+ vector for expression of common carp (*Cyprinus carpio*) corticosteroid-binding globulin-like isoform X1 (XP_018935807, now updated to XP_018935808.2)

| Species | Sex | Sample ID | Thiaminase activity (pmol/g/min) |
| --- | --- | --- | --- |
| *Danio rerio* | F | ZF-F-1 | 19,800 |
| *Danio rerio* | F | ZF-F-2 | 44,300 |
| *Danio rerio* | M | ZF-M-1 | 29,700 |
| *Danio rerio* | M | ZF-M-2 | 44,700 |

**Supplementary Table S1.** Thiaminase activity measured in whole zebrafish (*Danio rerio*) homogenates.

| Organism | Blast name | Score | Number of hits |
| --- | --- | --- | --- |
| [Clupeocephala](https://www.ncbi.nlm.nih.gov/Taxonomy/Browser/wwwtax.cgi?id=186625) | [bony fishes](https://www.ncbi.nlm.nih.gov/Taxonomy/Browser/wwwtax.cgi?id=7898) |  | [114](https://blast.ncbi.nlm.nih.gov/Blast.cgi) |
| .[Otomorpha](https://www.ncbi.nlm.nih.gov/Taxonomy/Browser/wwwtax.cgi?id=186634" \o "Show taxonomy info for Otomorpha (taxid 186634)" \t "lnktxRJF12C80013) | [bony fishes](https://www.ncbi.nlm.nih.gov/Taxonomy/Browser/wwwtax.cgi?id=7898) |  | [93](https://blast.ncbi.nlm.nih.gov/Blast.cgi) |
| ..[Ostariophysi](https://www.ncbi.nlm.nih.gov/Taxonomy/Browser/wwwtax.cgi?id=32519) | [bony fishes](https://www.ncbi.nlm.nih.gov/Taxonomy/Browser/wwwtax.cgi?id=7898) |  | [79](https://blast.ncbi.nlm.nih.gov/Blast.cgi) |
| ...[Otophysi](https://www.ncbi.nlm.nih.gov/Taxonomy/Browser/wwwtax.cgi?id=186626" \o "Show taxonomy info for Otophysi (taxid 186626)" \t "lnktxRJF12C80013) | [bony fishes](https://www.ncbi.nlm.nih.gov/Taxonomy/Browser/wwwtax.cgi?id=7898) |  | [77](https://blast.ncbi.nlm.nih.gov/Blast.cgi) |
| ....[Cypriniformes](https://www.ncbi.nlm.nih.gov/Taxonomy/Browser/wwwtax.cgi?id=7952" \o "Show taxonomy info for Cypriniformes (taxid 7952)" \t "lnktxRJF12C80013) | [bony fishes](https://www.ncbi.nlm.nih.gov/Taxonomy/Browser/wwwtax.cgi?id=7898) |  | [52](https://blast.ncbi.nlm.nih.gov/Blast.cgi) |
| .....[Cyprinoidei](https://www.ncbi.nlm.nih.gov/Taxonomy/Browser/wwwtax.cgi?id=30727" \o "Show taxonomy info for Cyprinoidei (taxid 30727)" \t "lnktxRJF12C80013) | [bony fishes](https://www.ncbi.nlm.nih.gov/Taxonomy/Browser/wwwtax.cgi?id=7898) |  | [48](https://blast.ncbi.nlm.nih.gov/Blast.cgi) |
| *......*[*Danio rerio*](https://www.ncbi.nlm.nih.gov/Taxonomy/Browser/wwwtax.cgi?id=7955) | [bony fishes](https://www.ncbi.nlm.nih.gov/Taxonomy/Browser/wwwtax.cgi?id=7898) | 464 | [2](https://blast.ncbi.nlm.nih.gov/Blast.cgi) |
| *......[Sinocyclocheilus rhinocerous](https://www.ncbi.nlm.nih.gov/Taxonomy/Browser/wwwtax.cgi?id=307959" \o "Show taxonomy info for Sinocyclocheilus rhinocerous (taxid 307959)" \t "lnktxRJF12C80013)* | [bony fishes](https://www.ncbi.nlm.nih.gov/Taxonomy/Browser/wwwtax.cgi?id=7898) | 343 | [2](https://blast.ncbi.nlm.nih.gov/Blast.cgi) |
| *......[Sinocyclocheilus grahami](https://www.ncbi.nlm.nih.gov/Taxonomy/Browser/wwwtax.cgi?id=75366" \o "Show taxonomy info for Sinocyclocheilus grahami (taxid 75366)" \t "lnktxRJF12C80013)* | [bony fishes](https://www.ncbi.nlm.nih.gov/Taxonomy/Browser/wwwtax.cgi?id=7898) | 338 | [1](https://blast.ncbi.nlm.nih.gov/Blast.cgi) |
| *......*[*Carassius auratus*](https://www.ncbi.nlm.nih.gov/Taxonomy/Browser/wwwtax.cgi?id=7957) | [bony fishes](https://www.ncbi.nlm.nih.gov/Taxonomy/Browser/wwwtax.cgi?id=7898) | 338 | [3](https://blast.ncbi.nlm.nih.gov/Blast.cgi) |
| *......*[*Cyprinus carpio*](https://www.ncbi.nlm.nih.gov/Taxonomy/Browser/wwwtax.cgi?id=7962) | [bony fishes](https://www.ncbi.nlm.nih.gov/Taxonomy/Browser/wwwtax.cgi?id=7898) | 338 | [7](https://blast.ncbi.nlm.nih.gov/Blast.cgi) |
| *......[Onychostoma macrolepis](https://www.ncbi.nlm.nih.gov/Taxonomy/Browser/wwwtax.cgi?id=369639" \o "Show taxonomy info for Onychostoma macrolepis (taxid 369639)" \t "lnktxRJF12C80013)* | [bony fishes](https://www.ncbi.nlm.nih.gov/Taxonomy/Browser/wwwtax.cgi?id=7898) | 338 | [1](https://blast.ncbi.nlm.nih.gov/Blast.cgi) |
| *......[Ctenopharyngodon idella](https://www.ncbi.nlm.nih.gov/Taxonomy/Browser/wwwtax.cgi?id=7959" \o "Show taxonomy info for Ctenopharyngodon idella (taxid 7959)" \t "lnktxRJF12C80013)* | [bony fishes](https://www.ncbi.nlm.nih.gov/Taxonomy/Browser/wwwtax.cgi?id=7898) | 335 | [5](https://blast.ncbi.nlm.nih.gov/Blast.cgi) |
| *......[Labeo rohita](https://www.ncbi.nlm.nih.gov/Taxonomy/Browser/wwwtax.cgi?id=84645" \o "Show taxonomy info for Labeo rohita (taxid 84645)" \t "lnktxRJF12C80013)* | [bony fishes](https://www.ncbi.nlm.nih.gov/Taxonomy/Browser/wwwtax.cgi?id=7898) | 334 | [10](https://blast.ncbi.nlm.nih.gov/Blast.cgi) |
| *......[Pimephales promelas](https://www.ncbi.nlm.nih.gov/Taxonomy/Browser/wwwtax.cgi?id=90988" \o "Show taxonomy info for Pimephales promelas (taxid 90988)" \t "lnktxRJF12C80013)* | [bony fishes](https://www.ncbi.nlm.nih.gov/Taxonomy/Browser/wwwtax.cgi?id=7898) | 333 | [7](https://blast.ncbi.nlm.nih.gov/Blast.cgi) |
| *......[Puntigrus tetrazona](https://www.ncbi.nlm.nih.gov/Taxonomy/Browser/wwwtax.cgi?id=1606681" \o "Show taxonomy info for Puntigrus tetrazona (taxid 1606681)" \t "lnktxRJF12C80013)* | [bony fishes](https://www.ncbi.nlm.nih.gov/Taxonomy/Browser/wwwtax.cgi?id=7898) | 322 | [2](https://blast.ncbi.nlm.nih.gov/Blast.cgi) |
| *......[Megalobrama amblycephala](https://www.ncbi.nlm.nih.gov/Taxonomy/Browser/wwwtax.cgi?id=75352" \o "Show taxonomy info for Megalobrama amblycephala (taxid 75352)" \t "lnktxRJF12C80013)* | [bony fishes](https://www.ncbi.nlm.nih.gov/Taxonomy/Browser/wwwtax.cgi?id=7898) | 321 | [3](https://blast.ncbi.nlm.nih.gov/Blast.cgi) |
| *......[Sinocyclocheilus anshuiensis](https://www.ncbi.nlm.nih.gov/Taxonomy/Browser/wwwtax.cgi?id=1608454" \o "Show taxonomy info for Sinocyclocheilus anshuiensis (taxid 1608454)" \t "lnktxRJF12C80013)* | [bony fishes](https://www.ncbi.nlm.nih.gov/Taxonomy/Browser/wwwtax.cgi?id=7898) | 321 | [1](https://blast.ncbi.nlm.nih.gov/Blast.cgi) |
| *......[Anabarilius grahami](https://www.ncbi.nlm.nih.gov/Taxonomy/Browser/wwwtax.cgi?id=495550" \o "Show taxonomy info for Anabarilius grahami (taxid 495550)" \t "lnktxRJF12C80013)* | [bony fishes](https://www.ncbi.nlm.nih.gov/Taxonomy/Browser/wwwtax.cgi?id=7898) | 314 | [4](https://blast.ncbi.nlm.nih.gov/Blast.cgi) |
| *.....[Myxocyprinus asiaticus](https://www.ncbi.nlm.nih.gov/Taxonomy/Browser/wwwtax.cgi?id=70543" \o "Show taxonomy info for Myxocyprinus asiaticus (taxid 70543)" \t "lnktxRJF12C80013)* | [bony fishes](https://www.ncbi.nlm.nih.gov/Taxonomy/Browser/wwwtax.cgi?id=7898) | 332 | [1](https://blast.ncbi.nlm.nih.gov/Blast.cgi) |
| *.....[Triplophysa tibetana](https://www.ncbi.nlm.nih.gov/Taxonomy/Browser/wwwtax.cgi?id=1572043" \o "Show taxonomy info for Triplophysa tibetana (taxid 1572043)" \t "lnktxRJF12C80013)* | [bony fishes](https://www.ncbi.nlm.nih.gov/Taxonomy/Browser/wwwtax.cgi?id=7898) | 324 | [1](https://blast.ncbi.nlm.nih.gov/Blast.cgi) |
| *.....[Triplophysa rosa](https://www.ncbi.nlm.nih.gov/Taxonomy/Browser/wwwtax.cgi?id=992332" \o "Show taxonomy info for Triplophysa rosa (taxid 992332)" \t "lnktxRJF12C80013)* | [bony fishes](https://www.ncbi.nlm.nih.gov/Taxonomy/Browser/wwwtax.cgi?id=7898) | 307 | [2](https://blast.ncbi.nlm.nih.gov/Blast.cgi) |
| *....[Pangasianodon hypophthalmus](https://www.ncbi.nlm.nih.gov/Taxonomy/Browser/wwwtax.cgi?id=310915" \o "Show taxonomy info for Pangasianodon hypophthalmus (taxid 310915)" \t "lnktxRJF12C80013)* | [bony fishes](https://www.ncbi.nlm.nih.gov/Taxonomy/Browser/wwwtax.cgi?id=7898) | 298 | [5](https://blast.ncbi.nlm.nih.gov/Blast.cgi) |
| *....[Colossoma macropomum](https://www.ncbi.nlm.nih.gov/Taxonomy/Browser/wwwtax.cgi?id=42526" \o "Show taxonomy info for Colossoma macropomum (taxid 42526)" \t "lnktxRJF12C80013)* | [bony fishes](https://www.ncbi.nlm.nih.gov/Taxonomy/Browser/wwwtax.cgi?id=7898) | 282 | [3](https://blast.ncbi.nlm.nih.gov/Blast.cgi) |
| *....[Hemibagrus wyckioides](https://www.ncbi.nlm.nih.gov/Taxonomy/Browser/wwwtax.cgi?id=337641" \o "Show taxonomy info for Hemibagrus wyckioides (taxid 337641)" \t "lnktxRJF12C80013)* | [bony fishes](https://www.ncbi.nlm.nih.gov/Taxonomy/Browser/wwwtax.cgi?id=7898) | 281 | [1](https://blast.ncbi.nlm.nih.gov/Blast.cgi) |
| *....[Tachysurus fulvidraco](https://www.ncbi.nlm.nih.gov/Taxonomy/Browser/wwwtax.cgi?id=1234273" \o "Show taxonomy info for Tachysurus fulvidraco (taxid 1234273)" \t "lnktxRJF12C80013)* | [bony fishes](https://www.ncbi.nlm.nih.gov/Taxonomy/Browser/wwwtax.cgi?id=7898) | 280 | [2](https://blast.ncbi.nlm.nih.gov/Blast.cgi) |
| *....[Pygocentrus nattereri](https://www.ncbi.nlm.nih.gov/Taxonomy/Browser/wwwtax.cgi?id=42514" \o "Show taxonomy info for Pygocentrus nattereri (taxid 42514)" \t "lnktxRJF12C80013)* | [bony fishes](https://www.ncbi.nlm.nih.gov/Taxonomy/Browser/wwwtax.cgi?id=7898) | 277 | [3](https://blast.ncbi.nlm.nih.gov/Blast.cgi) |
| *....[Prochilodus magdalenae](https://www.ncbi.nlm.nih.gov/Taxonomy/Browser/wwwtax.cgi?id=148989" \o "Show taxonomy info for Prochilodus magdalenae (taxid 148989)" \t "lnktxRJF12C80013)* | [bony fishes](https://www.ncbi.nlm.nih.gov/Taxonomy/Browser/wwwtax.cgi?id=7898) | 275 | [2](https://blast.ncbi.nlm.nih.gov/Blast.cgi) |
| *....*[*Astyanax mexicanus*](https://www.ncbi.nlm.nih.gov/Taxonomy/Browser/wwwtax.cgi?id=7994) | [bony fishes](https://www.ncbi.nlm.nih.gov/Taxonomy/Browser/wwwtax.cgi?id=7898) | 273 | [3](https://blast.ncbi.nlm.nih.gov/Blast.cgi) |
| *....*[*Ameiurus melas*](https://www.ncbi.nlm.nih.gov/Taxonomy/Browser/wwwtax.cgi?id=219545) | [bony fishes](https://www.ncbi.nlm.nih.gov/Taxonomy/Browser/wwwtax.cgi?id=7898) | 267 | [1](https://blast.ncbi.nlm.nih.gov/Blast.cgi) |
| *....[Silurus meridionalis](https://www.ncbi.nlm.nih.gov/Taxonomy/Browser/wwwtax.cgi?id=175797" \o "Show taxonomy info for Silurus meridionalis (taxid 175797)" \t "lnktxRJF12C80013)* | [bony fishes](https://www.ncbi.nlm.nih.gov/Taxonomy/Browser/wwwtax.cgi?id=7898) | 263 | [2](https://blast.ncbi.nlm.nih.gov/Blast.cgi) |
| *....*[*Electrophorus electricus*](https://www.ncbi.nlm.nih.gov/Taxonomy/Browser/wwwtax.cgi?id=8005) | [bony fishes](https://www.ncbi.nlm.nih.gov/Taxonomy/Browser/wwwtax.cgi?id=7898) | 253 | [1](https://blast.ncbi.nlm.nih.gov/Blast.cgi) |
| *....[Ictalurus punctatus](https://www.ncbi.nlm.nih.gov/Taxonomy/Browser/wwwtax.cgi?id=7998" \o "Show taxonomy info for Ictalurus punctatus (taxid 7998)" \t "lnktxRJF12C80013)* | [bony fishes](https://www.ncbi.nlm.nih.gov/Taxonomy/Browser/wwwtax.cgi?id=7898) | 251 | [1](https://blast.ncbi.nlm.nih.gov/Blast.cgi) |
| *....[Bagarius yarrelli](https://www.ncbi.nlm.nih.gov/Taxonomy/Browser/wwwtax.cgi?id=175774" \o "Show taxonomy info for Bagarius yarrelli (taxid 175774)" \t "lnktxRJF12C80013)* | [bony fishes](https://www.ncbi.nlm.nih.gov/Taxonomy/Browser/wwwtax.cgi?id=7898) | 239 | [1](https://blast.ncbi.nlm.nih.gov/Blast.cgi) |
| *...[Chanos chanos](https://www.ncbi.nlm.nih.gov/Taxonomy/Browser/wwwtax.cgi?id=29144" \o "Show taxonomy info for Chanos chanos (taxid 29144)" \t "lnktxRJF12C80013)* | [bony fishes](https://www.ncbi.nlm.nih.gov/Taxonomy/Browser/wwwtax.cgi?id=7898) | 271 | [2](https://blast.ncbi.nlm.nih.gov/Blast.cgi) |
| *..*[*Alosa sapidissima*](https://www.ncbi.nlm.nih.gov/Taxonomy/Browser/wwwtax.cgi?id=34773) | [bony fishes](https://www.ncbi.nlm.nih.gov/Taxonomy/Browser/wwwtax.cgi?id=7898) | 254 | [4](https://blast.ncbi.nlm.nih.gov/Blast.cgi) |
| *..*[*Alosa alosa*](https://www.ncbi.nlm.nih.gov/Taxonomy/Browser/wwwtax.cgi?id=278164) | [bony fishes](https://www.ncbi.nlm.nih.gov/Taxonomy/Browser/wwwtax.cgi?id=7898) | 251 | [4](https://blast.ncbi.nlm.nih.gov/Blast.cgi) |
| *..*[*Clupea harengus*](https://www.ncbi.nlm.nih.gov/Taxonomy/Browser/wwwtax.cgi?id=7950) | [bony fishes](https://www.ncbi.nlm.nih.gov/Taxonomy/Browser/wwwtax.cgi?id=7898) | 222 | [5](https://blast.ncbi.nlm.nih.gov/Blast.cgi) |
| *..[Denticeps clupeoides](https://www.ncbi.nlm.nih.gov/Taxonomy/Browser/wwwtax.cgi?id=299321" \o "Show taxonomy info for Denticeps clupeoides (taxid 299321)" \t "lnktxRJF12C80013)* | [bony fishes](https://www.ncbi.nlm.nih.gov/Taxonomy/Browser/wwwtax.cgi?id=7898) | 210 | [1](https://blast.ncbi.nlm.nih.gov/Blast.cgi) |
| *.[Fundulus heteroclitus](https://www.ncbi.nlm.nih.gov/Taxonomy/Browser/wwwtax.cgi?id=8078" \o "Show taxonomy info for Fundulus heteroclitus (taxid 8078)" \t "lnktxRJF12C80013)* | [bony fishes](https://www.ncbi.nlm.nih.gov/Taxonomy/Browser/wwwtax.cgi?id=7898) | 228 | [2](https://blast.ncbi.nlm.nih.gov/Blast.cgi) |
| *.[Cyprinodon tularosa](https://www.ncbi.nlm.nih.gov/Taxonomy/Browser/wwwtax.cgi?id=77115" \o "Show taxonomy info for Cyprinodon tularosa (taxid 77115)" \t "lnktxRJF12C80013)* | [bony fishes](https://www.ncbi.nlm.nih.gov/Taxonomy/Browser/wwwtax.cgi?id=7898) | 226 | [1](https://blast.ncbi.nlm.nih.gov/Blast.cgi) |
| *.*[*Mugil cephalus*](https://www.ncbi.nlm.nih.gov/Taxonomy/Browser/wwwtax.cgi?id=48193) | [bony fishes](https://www.ncbi.nlm.nih.gov/Taxonomy/Browser/wwwtax.cgi?id=7898) | 223 | [1](https://blast.ncbi.nlm.nih.gov/Blast.cgi) |
| *.*[*Xiphophorus hellerii*](https://www.ncbi.nlm.nih.gov/Taxonomy/Browser/wwwtax.cgi?id=8084) | [bony fishes](https://www.ncbi.nlm.nih.gov/Taxonomy/Browser/wwwtax.cgi?id=7898) | 216 | [1](https://blast.ncbi.nlm.nih.gov/Blast.cgi) |
| *.[Neolamprologus brichardi](https://www.ncbi.nlm.nih.gov/Taxonomy/Browser/wwwtax.cgi?id=32507" \o "Show taxonomy info for Neolamprologus brichardi (taxid 32507)" \t "lnktxRJF12C80013)* | [bony fishes](https://www.ncbi.nlm.nih.gov/Taxonomy/Browser/wwwtax.cgi?id=7898) | 215 | [1](https://blast.ncbi.nlm.nih.gov/Blast.cgi) |
| *.[Menidia menidia](https://www.ncbi.nlm.nih.gov/Taxonomy/Browser/wwwtax.cgi?id=238744" \o "Show taxonomy info for Menidia menidia (taxid 238744)" \t "lnktxRJF12C80013)* | [bony fishes](https://www.ncbi.nlm.nih.gov/Taxonomy/Browser/wwwtax.cgi?id=7898) | 224 | [2](https://blast.ncbi.nlm.nih.gov/Blast.cgi) |
| *.*[*Oreochromis niloticus*](https://www.ncbi.nlm.nih.gov/Taxonomy/Browser/wwwtax.cgi?id=8128) | [bony fishes](https://www.ncbi.nlm.nih.gov/Taxonomy/Browser/wwwtax.cgi?id=7898) | 212 | [1](https://blast.ncbi.nlm.nih.gov/Blast.cgi) |
| *.*[*Oreochromis aureus*](https://www.ncbi.nlm.nih.gov/Taxonomy/Browser/wwwtax.cgi?id=47969) | [bony fishes](https://www.ncbi.nlm.nih.gov/Taxonomy/Browser/wwwtax.cgi?id=7898) | 212 | [2](https://blast.ncbi.nlm.nih.gov/Blast.cgi) |
| *.*[*Xiphophorus maculatus*](https://www.ncbi.nlm.nih.gov/Taxonomy/Browser/wwwtax.cgi?id=8083) | [bony fishes](https://www.ncbi.nlm.nih.gov/Taxonomy/Browser/wwwtax.cgi?id=7898) | 209 | [1](https://blast.ncbi.nlm.nih.gov/Blast.cgi) |
| *.[Poecilia latipinna](https://www.ncbi.nlm.nih.gov/Taxonomy/Browser/wwwtax.cgi?id=48699" \o "Show taxonomy info for Poecilia latipinna (taxid 48699)" \t "lnktxRJF12C80013)* | [bony fishes](https://www.ncbi.nlm.nih.gov/Taxonomy/Browser/wwwtax.cgi?id=7898) | 207 | [2](https://blast.ncbi.nlm.nih.gov/Blast.cgi) |
| *.[Poecilia formosa](https://www.ncbi.nlm.nih.gov/Taxonomy/Browser/wwwtax.cgi?id=48698" \o "Show taxonomy info for Poecilia formosa (taxid 48698)" \t "lnktxRJF12C80013)* | [bony fishes](https://www.ncbi.nlm.nih.gov/Taxonomy/Browser/wwwtax.cgi?id=7898) | 208 | [3](https://blast.ncbi.nlm.nih.gov/Blast.cgi) |
| *.[Poecilia mexicana](https://www.ncbi.nlm.nih.gov/Taxonomy/Browser/wwwtax.cgi?id=48701" \o "Show taxonomy info for Poecilia mexicana (taxid 48701)" \t "lnktxRJF12C80013)* | [bony fishes](https://www.ncbi.nlm.nih.gov/Taxonomy/Browser/wwwtax.cgi?id=7898) | 208 | [2](https://blast.ncbi.nlm.nih.gov/Blast.cgi) |
| *.*[*Gambusia affinis*](https://www.ncbi.nlm.nih.gov/Taxonomy/Browser/wwwtax.cgi?id=33528) | [bony fishes](https://www.ncbi.nlm.nih.gov/Taxonomy/Browser/wwwtax.cgi?id=7898) | 206 | [1](https://blast.ncbi.nlm.nih.gov/Blast.cgi) |
| *.[Haplochromis burtoni](https://www.ncbi.nlm.nih.gov/Taxonomy/Browser/wwwtax.cgi?id=8153" \o "Show taxonomy info for Haplochromis burtoni (taxid 8153)" \t "lnktxRJF12C80013)* | [bony fishes](https://www.ncbi.nlm.nih.gov/Taxonomy/Browser/wwwtax.cgi?id=7898) | 202 | [1](https://blast.ncbi.nlm.nih.gov/Blast.cgi) |

**Supplementary Table S2.** Taxonomy report for an unrestricted BLASTP search of the GenBank nr protein database with putative zebrafish (*Danio rerio*) thiaminase I sequence [NP_001314821.1](https://www.ncbi.nlm.nih.gov/sites/entrez?cmd=Search&db=protein&term=NP_001314821.1&dopt=GenBank), Max target sequences = 100.

| Organism | Blast name | Score | Number of hits |
| --- | --- | --- | --- |
| [Eukaryota](https://www.ncbi.nlm.nih.gov/Taxonomy/Browser/wwwtax.cgi?id=2759) | [eukaryotes](https://www.ncbi.nlm.nih.gov/Taxonomy/Browser/wwwtax.cgi?id=2759) |  | [126](https://blast.ncbi.nlm.nih.gov/Blast.cgi) |
| .[Eumetazoa](https://www.ncbi.nlm.nih.gov/Taxonomy/Browser/wwwtax.cgi?id=6072) | [animals](https://www.ncbi.nlm.nih.gov/Taxonomy/Browser/wwwtax.cgi?id=33208) |  | [104](https://blast.ncbi.nlm.nih.gov/Blast.cgi) |
| ..[Bilateria](https://www.ncbi.nlm.nih.gov/Taxonomy/Browser/wwwtax.cgi?id=33213) | [animals](https://www.ncbi.nlm.nih.gov/Taxonomy/Browser/wwwtax.cgi?id=33208) |  | [69](https://blast.ncbi.nlm.nih.gov/Blast.cgi) |
| ...[Deuterostomia](https://www.ncbi.nlm.nih.gov/Taxonomy/Browser/wwwtax.cgi?id=33511) | [animals](https://www.ncbi.nlm.nih.gov/Taxonomy/Browser/wwwtax.cgi?id=33208) |  | [22](https://blast.ncbi.nlm.nih.gov/Blast.cgi) |
| ....[Chordata](https://www.ncbi.nlm.nih.gov/Taxonomy/Browser/wwwtax.cgi?id=7711) | [chordates](https://www.ncbi.nlm.nih.gov/Taxonomy/Browser/wwwtax.cgi?id=7711) |  | [10](https://blast.ncbi.nlm.nih.gov/Blast.cgi) |
| *.....[Protopterus annectens](https://www.ncbi.nlm.nih.gov/Taxonomy/Browser/wwwtax.cgi?id=7888" \o "Show taxonomy info for Protopterus annectens (taxid 7888)" \t "lnktxRJF4YDRC013)* | [lungfishes](https://www.ncbi.nlm.nih.gov/Taxonomy/Browser/wwwtax.cgi?id=7878) | 162 | [6](https://blast.ncbi.nlm.nih.gov/Blast.cgi) |
| *.....[Branchiostoma lanceolatum](https://www.ncbi.nlm.nih.gov/Taxonomy/Browser/wwwtax.cgi?id=7740" \o "Show taxonomy info for Branchiostoma lanceolatum (taxid 7740)" \t "lnktxRJF4YDRC013)* | [lancelets](https://www.ncbi.nlm.nih.gov/Taxonomy/Browser/wwwtax.cgi?id=7735) | 89.0 | [1](https://blast.ncbi.nlm.nih.gov/Blast.cgi) |
| *.....[Branchiostoma belcheri](https://www.ncbi.nlm.nih.gov/Taxonomy/Browser/wwwtax.cgi?id=7741" \o "Show taxonomy info for Branchiostoma belcheri (taxid 7741)" \t "lnktxRJF4YDRC013)* | [lancelets](https://www.ncbi.nlm.nih.gov/Taxonomy/Browser/wwwtax.cgi?id=7735) | 84.0 | [2](https://blast.ncbi.nlm.nih.gov/Blast.cgi) |
| *.....[Branchiostoma floridae](https://www.ncbi.nlm.nih.gov/Taxonomy/Browser/wwwtax.cgi?id=7739" \o "Show taxonomy info for Branchiostoma floridae (taxid 7739)" \t "lnktxRJF4YDRC013)* | [lancelets](https://www.ncbi.nlm.nih.gov/Taxonomy/Browser/wwwtax.cgi?id=7735) | 55.1 | [1](https://blast.ncbi.nlm.nih.gov/Blast.cgi) |
| *....*[*Strongylocentrotus purpuratus*](https://www.ncbi.nlm.nih.gov/Taxonomy/Browser/wwwtax.cgi?id=7668) | [sea urchins](https://www.ncbi.nlm.nih.gov/Taxonomy/Browser/wwwtax.cgi?id=7625) | 94.7 | [7](https://blast.ncbi.nlm.nih.gov/Blast.cgi) |
| *....[Patiria miniata](https://www.ncbi.nlm.nih.gov/Taxonomy/Browser/wwwtax.cgi?id=46514" \o "Show taxonomy info for Patiria miniata (taxid 46514)" \t "lnktxRJF4YDRC013)* | [starfish](https://www.ncbi.nlm.nih.gov/Taxonomy/Browser/wwwtax.cgi?id=7588) | 89.4 | [3](https://blast.ncbi.nlm.nih.gov/Blast.cgi) |
| *....[Acanthaster planci](https://www.ncbi.nlm.nih.gov/Taxonomy/Browser/wwwtax.cgi?id=133434" \o "Show taxonomy info for Acanthaster planci (taxid 133434)" \t "lnktxRJF4YDRC013)* | [starfish](https://www.ncbi.nlm.nih.gov/Taxonomy/Browser/wwwtax.cgi?id=7588) | 89.0 | [2](https://blast.ncbi.nlm.nih.gov/Blast.cgi) |
| *...[Mizuhopecten yessoensis](https://www.ncbi.nlm.nih.gov/Taxonomy/Browser/wwwtax.cgi?id=6573" \o "Show taxonomy info for Mizuhopecten yessoensis (taxid 6573)" \t "lnktxRJF4YDRC013)* | [bivalves](https://www.ncbi.nlm.nih.gov/Taxonomy/Browser/wwwtax.cgi?id=6544) | 81.3 | [19](https://blast.ncbi.nlm.nih.gov/Blast.cgi) |
| *...*[*Mytilus coruscus*](https://www.ncbi.nlm.nih.gov/Taxonomy/Browser/wwwtax.cgi?id=42192) | [bivalves](https://www.ncbi.nlm.nih.gov/Taxonomy/Browser/wwwtax.cgi?id=6544) | 78.2 | [5](https://blast.ncbi.nlm.nih.gov/Blast.cgi) |
| *...*[*Pecten maximus*](https://www.ncbi.nlm.nih.gov/Taxonomy/Browser/wwwtax.cgi?id=6579) | [bivalves](https://www.ncbi.nlm.nih.gov/Taxonomy/Browser/wwwtax.cgi?id=6544) | 75.5 | [4](https://blast.ncbi.nlm.nih.gov/Blast.cgi) |
| *...*[*Mercenaria mercenaria*](https://www.ncbi.nlm.nih.gov/Taxonomy/Browser/wwwtax.cgi?id=6596) | [bivalves](https://www.ncbi.nlm.nih.gov/Taxonomy/Browser/wwwtax.cgi?id=6544) | 75.5 | [9](https://blast.ncbi.nlm.nih.gov/Blast.cgi) |
| *...*[*Mytilus galloprovincialis*](https://www.ncbi.nlm.nih.gov/Taxonomy/Browser/wwwtax.cgi?id=29158) | [bivalves](https://www.ncbi.nlm.nih.gov/Taxonomy/Browser/wwwtax.cgi?id=6544) | 74.7 | [6](https://blast.ncbi.nlm.nih.gov/Blast.cgi) |
| *...*[*Mytilus edulis*](https://www.ncbi.nlm.nih.gov/Taxonomy/Browser/wwwtax.cgi?id=6550) | [bivalves](https://www.ncbi.nlm.nih.gov/Taxonomy/Browser/wwwtax.cgi?id=6544) | 65.5 | [1](https://blast.ncbi.nlm.nih.gov/Blast.cgi) |
| *...[Dreissena polymorpha](https://www.ncbi.nlm.nih.gov/Taxonomy/Browser/wwwtax.cgi?id=45954" \o "Show taxonomy info for Dreissena polymorpha (taxid 45954)" \t "lnktxRJF4YDRC013)* | [bivalves](https://www.ncbi.nlm.nih.gov/Taxonomy/Browser/wwwtax.cgi?id=6544) | 65.5 | [3](https://blast.ncbi.nlm.nih.gov/Blast.cgi) |
| *..[Dendronephthya gigantea](https://www.ncbi.nlm.nih.gov/Taxonomy/Browser/wwwtax.cgi?id=151771" \o "Show taxonomy info for Dendronephthya gigantea (taxid 151771)" \t "lnktxRJF4YDRC013)* | [soft corals](https://www.ncbi.nlm.nih.gov/Taxonomy/Browser/wwwtax.cgi?id=40677) | 87.8 | [3](https://blast.ncbi.nlm.nih.gov/Blast.cgi) |
| *..[Paramuricea clavata](https://www.ncbi.nlm.nih.gov/Taxonomy/Browser/wwwtax.cgi?id=317549" \o "Show taxonomy info for Paramuricea clavata (taxid 317549)" \t "lnktxRJF4YDRC013)* | [soft corals](https://www.ncbi.nlm.nih.gov/Taxonomy/Browser/wwwtax.cgi?id=40677) | 84.3 | [3](https://blast.ncbi.nlm.nih.gov/Blast.cgi) |
| *..*[*Acropora digitifera*](https://www.ncbi.nlm.nih.gov/Taxonomy/Browser/wwwtax.cgi?id=70779) | [stony corals](https://www.ncbi.nlm.nih.gov/Taxonomy/Browser/wwwtax.cgi?id=6125) | 77.8 | [6](https://blast.ncbi.nlm.nih.gov/Blast.cgi) |
| *..*[*Xenia sp. Carnegie-2017*](https://www.ncbi.nlm.nih.gov/Taxonomy/Browser/wwwtax.cgi?id=2897299) | [soft corals](https://www.ncbi.nlm.nih.gov/Taxonomy/Browser/wwwtax.cgi?id=40677) | 75.5 | [1](https://blast.ncbi.nlm.nih.gov/Blast.cgi) |
| *..*[*Acropora millepora*](https://www.ncbi.nlm.nih.gov/Taxonomy/Browser/wwwtax.cgi?id=45264) | [stony corals](https://www.ncbi.nlm.nih.gov/Taxonomy/Browser/wwwtax.cgi?id=6125) | 74.7 | [14](https://blast.ncbi.nlm.nih.gov/Blast.cgi) |
| *..[Pocillopora damicornis](https://www.ncbi.nlm.nih.gov/Taxonomy/Browser/wwwtax.cgi?id=46731" \o "Show taxonomy info for Pocillopora damicornis (taxid 46731)" \t "lnktxRJF4YDRC013)* | [stony corals](https://www.ncbi.nlm.nih.gov/Taxonomy/Browser/wwwtax.cgi?id=6125) | 70.5 | [3](https://blast.ncbi.nlm.nih.gov/Blast.cgi) |
| *..[Orbicella faveolata](https://www.ncbi.nlm.nih.gov/Taxonomy/Browser/wwwtax.cgi?id=48498" \o "Show taxonomy info for Orbicella faveolata (taxid 48498)" \t "lnktxRJF4YDRC013)* | [stony corals](https://www.ncbi.nlm.nih.gov/Taxonomy/Browser/wwwtax.cgi?id=6125) | 68.6 | [3](https://blast.ncbi.nlm.nih.gov/Blast.cgi) |
| *..[Exaiptasia diaphana](https://www.ncbi.nlm.nih.gov/Taxonomy/Browser/wwwtax.cgi?id=2652724" \o "Show taxonomy info for Exaiptasia diaphana (taxid 2652724)" \t "lnktxRJF4YDRC013)* | [sea anemones](https://www.ncbi.nlm.nih.gov/Taxonomy/Browser/wwwtax.cgi?id=6103) | 61.2 | [2](https://blast.ncbi.nlm.nih.gov/Blast.cgi) |
| *.[Gracilariopsis chorda](https://www.ncbi.nlm.nih.gov/Taxonomy/Browser/wwwtax.cgi?id=448386" \o "Show taxonomy info for Gracilariopsis chorda (taxid 448386)" \t "lnktxRJF4YDRC013)* | [red algae](https://www.ncbi.nlm.nih.gov/Taxonomy/Browser/wwwtax.cgi?id=2763) | 63.5 | [1](https://blast.ncbi.nlm.nih.gov/Blast.cgi) |
| *.[Perkinsus olseni](https://www.ncbi.nlm.nih.gov/Taxonomy/Browser/wwwtax.cgi?id=32597" \o "Show taxonomy info for Perkinsus olseni (taxid 32597)" \t "lnktxRJF4YDRC013)* | [eukaryotes](https://www.ncbi.nlm.nih.gov/Taxonomy/Browser/wwwtax.cgi?id=2759) | 63.5 | [14](https://blast.ncbi.nlm.nih.gov/Blast.cgi) |
| *.[Perkinsus marinus atcc 50983](https://www.ncbi.nlm.nih.gov/Taxonomy/Browser/wwwtax.cgi?id=423536" \o "Show taxonomy info for Perkinsus marinus ATCC 50983 (taxid 423536)" \t "lnktxRJF4YDRC013)* | [eukaryotes](https://www.ncbi.nlm.nih.gov/Taxonomy/Browser/wwwtax.cgi?id=2759) | 60.8 | [4](https://blast.ncbi.nlm.nih.gov/Blast.cgi) |
| *.*[*Chondrus crispus*](https://www.ncbi.nlm.nih.gov/Taxonomy/Browser/wwwtax.cgi?id=2769) | [red algae](https://www.ncbi.nlm.nih.gov/Taxonomy/Browser/wwwtax.cgi?id=2763) | 56.6 | [2](https://blast.ncbi.nlm.nih.gov/Blast.cgi) |
| *.[Auxenochlorella protothecoides](https://www.ncbi.nlm.nih.gov/Taxonomy/Browser/wwwtax.cgi?id=3075" \o "Show taxonomy info for Auxenochlorella protothecoides (taxid 3075)" \t "lnktxRJF4YDRC013)* | [green algae](https://www.ncbi.nlm.nih.gov/Taxonomy/Browser/wwwtax.cgi?id=3041) | 53.5 | [1](https://blast.ncbi.nlm.nih.gov/Blast.cgi) |

**Supplementary Table S3.** Taxonomy report for an BLASTP search of the GenBank nr protein database limited to include Eukaryota (taxid:2759) and exclude bony fish (taxid:7898) with putative zebrafish (*Danio rerio*) thiaminase I sequence [NP_001314821.1](https://www.ncbi.nlm.nih.gov/sites/entrez?cmd=Search&db=protein&term=NP_001314821.1&dopt=GenBank), Max target sequences = 100.

| Organism | Blast name | Score | Number of hits |
| --- | --- | --- | --- |
| [Bacteria](https://www.ncbi.nlm.nih.gov/Taxonomy/Browser/wwwtax.cgi?id=2) | [bacteria](https://www.ncbi.nlm.nih.gov/Taxonomy/Browser/wwwtax.cgi?id=-1) |  | [261](https://blast.ncbi.nlm.nih.gov/Blast.cgi) |
| .[Proteobacteria](https://www.ncbi.nlm.nih.gov/Taxonomy/Browser/wwwtax.cgi?id=1224) | [proteobacteria](https://www.ncbi.nlm.nih.gov/Taxonomy/Browser/wwwtax.cgi?id=1224) |  | [165](https://blast.ncbi.nlm.nih.gov/Blast.cgi) |
| ..[Stigmatella](https://www.ncbi.nlm.nih.gov/Taxonomy/Browser/wwwtax.cgi?id=40) | [d-proteobacteria](https://www.ncbi.nlm.nih.gov/Taxonomy/Browser/wwwtax.cgi?id=28221) |  | [3](https://blast.ncbi.nlm.nih.gov/Blast.cgi) |
| ...[Stigmatella aurantiaca](https://www.ncbi.nlm.nih.gov/Taxonomy/Browser/wwwtax.cgi?id=41) | [d-proteobacteria](https://www.ncbi.nlm.nih.gov/Taxonomy/Browser/wwwtax.cgi?id=28221) | 90.5 | [1](https://blast.ncbi.nlm.nih.gov/Blast.cgi) |
| ...[Stigmatella aurantiaca DW4/3-1](https://www.ncbi.nlm.nih.gov/Taxonomy/Browser/wwwtax.cgi?id=378806) | [d-proteobacteria](https://www.ncbi.nlm.nih.gov/Taxonomy/Browser/wwwtax.cgi?id=28221) | 90.5 | [2](https://blast.ncbi.nlm.nih.gov/Blast.cgi) |
| ..[Burkholderia pseudomallei](https://www.ncbi.nlm.nih.gov/Taxonomy/Browser/wwwtax.cgi?id=28450) | [b-proteobacteria](https://www.ncbi.nlm.nih.gov/Taxonomy/Browser/wwwtax.cgi?id=28216) | 80.5 | [71](https://blast.ncbi.nlm.nih.gov/Blast.cgi) |
| ..[Burkholderia pseudomallei 1655](https://www.ncbi.nlm.nih.gov/Taxonomy/Browser/wwwtax.cgi?id=331109) | [b-proteobacteria](https://www.ncbi.nlm.nih.gov/Taxonomy/Browser/wwwtax.cgi?id=28216) | 79.0 | [1](https://blast.ncbi.nlm.nih.gov/Blast.cgi) |
| ..[Burkholderia](https://www.ncbi.nlm.nih.gov/Taxonomy/Browser/wwwtax.cgi?id=32008) | [b-proteobacteria](https://www.ncbi.nlm.nih.gov/Taxonomy/Browser/wwwtax.cgi?id=28216) | 78.6 | [3](https://blast.ncbi.nlm.nih.gov/Blast.cgi) |
| ..[Burkholderia pseudomallei MSHR5858](https://www.ncbi.nlm.nih.gov/Taxonomy/Browser/wwwtax.cgi?id=1306417) | [b-proteobacteria](https://www.ncbi.nlm.nih.gov/Taxonomy/Browser/wwwtax.cgi?id=28216) | 78.6 | [1](https://blast.ncbi.nlm.nih.gov/Blast.cgi) |
| ..[Burkholderia pseudomallei PB08298010](https://www.ncbi.nlm.nih.gov/Taxonomy/Browser/wwwtax.cgi?id=1249658) | [b-proteobacteria](https://www.ncbi.nlm.nih.gov/Taxonomy/Browser/wwwtax.cgi?id=28216) | 78.6 | [1](https://blast.ncbi.nlm.nih.gov/Blast.cgi) |
| ..[Burkholderia pseudomallei MSHR2243](https://www.ncbi.nlm.nih.gov/Taxonomy/Browser/wwwtax.cgi?id=1435994) | [b-proteobacteria](https://www.ncbi.nlm.nih.gov/Taxonomy/Browser/wwwtax.cgi?id=28216) | 78.6 | [1](https://blast.ncbi.nlm.nih.gov/Blast.cgi) |
| ..[Burkholderia pseudomallei MSHR3965](https://www.ncbi.nlm.nih.gov/Taxonomy/Browser/wwwtax.cgi?id=1435372) | [b-proteobacteria](https://www.ncbi.nlm.nih.gov/Taxonomy/Browser/wwwtax.cgi?id=28216) | 78.6 | [1](https://blast.ncbi.nlm.nih.gov/Blast.cgi) |
| ..[Burkholderia pseudomallei MSHR2543](https://www.ncbi.nlm.nih.gov/Taxonomy/Browser/wwwtax.cgi?id=1249472) | [b-proteobacteria](https://www.ncbi.nlm.nih.gov/Taxonomy/Browser/wwwtax.cgi?id=28216) | 78.6 | [1](https://blast.ncbi.nlm.nih.gov/Blast.cgi) |
| ..[Burkholderia pseudomallei MSHR1029](https://www.ncbi.nlm.nih.gov/Taxonomy/Browser/wwwtax.cgi?id=1435991) | [b-proteobacteria](https://www.ncbi.nlm.nih.gov/Taxonomy/Browser/wwwtax.cgi?id=28216) | 78.6 | [1](https://blast.ncbi.nlm.nih.gov/Blast.cgi) |
| ..[Burkholderia pseudomallei MSHR3458](https://www.ncbi.nlm.nih.gov/Taxonomy/Browser/wwwtax.cgi?id=1437003) | [b-proteobacteria](https://www.ncbi.nlm.nih.gov/Taxonomy/Browser/wwwtax.cgi?id=28216) | 78.6 | [1](https://blast.ncbi.nlm.nih.gov/Blast.cgi) |
| ..[Burkholderia pseudomallei TSV32](https://www.ncbi.nlm.nih.gov/Taxonomy/Browser/wwwtax.cgi?id=1439859) | [b-proteobacteria](https://www.ncbi.nlm.nih.gov/Taxonomy/Browser/wwwtax.cgi?id=28216) | 78.6 | [1](https://blast.ncbi.nlm.nih.gov/Blast.cgi) |
| ..[Burkholderia pseudomallei NCTC 13179](https://www.ncbi.nlm.nih.gov/Taxonomy/Browser/wwwtax.cgi?id=1241583) | [b-proteobacteria](https://www.ncbi.nlm.nih.gov/Taxonomy/Browser/wwwtax.cgi?id=28216) | 78.6 | [1](https://blast.ncbi.nlm.nih.gov/Blast.cgi) |
| ..[Burkholderia pseudomallei NCTC 13178](https://www.ncbi.nlm.nih.gov/Taxonomy/Browser/wwwtax.cgi?id=1249468) | [b-proteobacteria](https://www.ncbi.nlm.nih.gov/Taxonomy/Browser/wwwtax.cgi?id=28216) | 78.6 | [1](https://blast.ncbi.nlm.nih.gov/Blast.cgi) |
| ..[Burkholderia pseudomallei MSHR1153](https://www.ncbi.nlm.nih.gov/Taxonomy/Browser/wwwtax.cgi?id=1437000) | [b-proteobacteria](https://www.ncbi.nlm.nih.gov/Taxonomy/Browser/wwwtax.cgi?id=28216) | 77.0 | [1](https://blast.ncbi.nlm.nih.gov/Blast.cgi) |
| ..[pseudomallei group](https://www.ncbi.nlm.nih.gov/Taxonomy/Browser/wwwtax.cgi?id=111527) | [b-proteobacteria](https://www.ncbi.nlm.nih.gov/Taxonomy/Browser/wwwtax.cgi?id=28216) | 76.6 | [1](https://blast.ncbi.nlm.nih.gov/Blast.cgi) |
| ..[Burkholderia pseudomallei CS](https://www.ncbi.nlm.nih.gov/Taxonomy/Browser/wwwtax.cgi?id=1290412) | [b-proteobacteria](https://www.ncbi.nlm.nih.gov/Taxonomy/Browser/wwwtax.cgi?id=28216) | 75.1 | [1](https://blast.ncbi.nlm.nih.gov/Blast.cgi) |
| ..[Burkholderia pseudomallei CB](https://www.ncbi.nlm.nih.gov/Taxonomy/Browser/wwwtax.cgi?id=1290413) | [b-proteobacteria](https://www.ncbi.nlm.nih.gov/Taxonomy/Browser/wwwtax.cgi?id=28216) | 75.1 | [1](https://blast.ncbi.nlm.nih.gov/Blast.cgi) |
| ..[Burkholderia pseudomallei OS](https://www.ncbi.nlm.nih.gov/Taxonomy/Browser/wwwtax.cgi?id=1290414) | [b-proteobacteria](https://www.ncbi.nlm.nih.gov/Taxonomy/Browser/wwwtax.cgi?id=28216) | 75.1 | [1](https://blast.ncbi.nlm.nih.gov/Blast.cgi) |
| ..[Burkholderia pseudomallei OB](https://www.ncbi.nlm.nih.gov/Taxonomy/Browser/wwwtax.cgi?id=1290415) | [b-proteobacteria](https://www.ncbi.nlm.nih.gov/Taxonomy/Browser/wwwtax.cgi?id=28216) | 75.1 | [1](https://blast.ncbi.nlm.nih.gov/Blast.cgi) |
| ..[Burkholderia sp. MSMB1498](https://www.ncbi.nlm.nih.gov/Taxonomy/Browser/wwwtax.cgi?id=1637842) | [b-proteobacteria](https://www.ncbi.nlm.nih.gov/Taxonomy/Browser/wwwtax.cgi?id=28216) | 74.3 | [1](https://blast.ncbi.nlm.nih.gov/Blast.cgi) |
| ..[Burkholderia sp. BDU5](https://www.ncbi.nlm.nih.gov/Taxonomy/Browser/wwwtax.cgi?id=1385590) | [b-proteobacteria](https://www.ncbi.nlm.nih.gov/Taxonomy/Browser/wwwtax.cgi?id=28216) | 73.9 | [1](https://blast.ncbi.nlm.nih.gov/Blast.cgi) |
| ..[Burkholderia oklahomensis](https://www.ncbi.nlm.nih.gov/Taxonomy/Browser/wwwtax.cgi?id=342113) | [b-proteobacteria](https://www.ncbi.nlm.nih.gov/Taxonomy/Browser/wwwtax.cgi?id=28216) | 72.4 | [1](https://blast.ncbi.nlm.nih.gov/Blast.cgi) |
| ..[Burkholderia sp. MSMB1589WGS](https://www.ncbi.nlm.nih.gov/Taxonomy/Browser/wwwtax.cgi?id=1636425) | [b-proteobacteria](https://www.ncbi.nlm.nih.gov/Taxonomy/Browser/wwwtax.cgi?id=28216) | 72.0 | [1](https://blast.ncbi.nlm.nih.gov/Blast.cgi) |
| ..[Rhodophyticola porphyridii](https://www.ncbi.nlm.nih.gov/Taxonomy/Browser/wwwtax.cgi?id=1852017) | [a-proteobacteria](https://www.ncbi.nlm.nih.gov/Taxonomy/Browser/wwwtax.cgi?id=28211) | 71.2 | [2](https://blast.ncbi.nlm.nih.gov/Blast.cgi) |
| ..[Burkholderia mayonis](https://www.ncbi.nlm.nih.gov/Taxonomy/Browser/wwwtax.cgi?id=1385591) | [b-proteobacteria](https://www.ncbi.nlm.nih.gov/Taxonomy/Browser/wwwtax.cgi?id=28216) | 69.3 | [1](https://blast.ncbi.nlm.nih.gov/Blast.cgi) |
| ..[Aeromonas rivuli](https://www.ncbi.nlm.nih.gov/Taxonomy/Browser/wwwtax.cgi?id=648794) | [g-proteobacteria](https://www.ncbi.nlm.nih.gov/Taxonomy/Browser/wwwtax.cgi?id=1236) | 68.9 | [3](https://blast.ncbi.nlm.nih.gov/Blast.cgi) |
| ..[unclassified Aeromonas](https://www.ncbi.nlm.nih.gov/Taxonomy/Browser/wwwtax.cgi?id=257493) | [g-proteobacteria](https://www.ncbi.nlm.nih.gov/Taxonomy/Browser/wwwtax.cgi?id=1236) | 68.9 | [1](https://blast.ncbi.nlm.nih.gov/Blast.cgi) |
| ..[Aeromonas sp. BIGb0405](https://www.ncbi.nlm.nih.gov/Taxonomy/Browser/wwwtax.cgi?id=2940592) | [g-proteobacteria](https://www.ncbi.nlm.nih.gov/Taxonomy/Browser/wwwtax.cgi?id=1236) | 68.9 | [1](https://blast.ncbi.nlm.nih.gov/Blast.cgi) |
| ..[Aeromonas sp. BIGb0445](https://www.ncbi.nlm.nih.gov/Taxonomy/Browser/wwwtax.cgi?id=2940593) | [g-proteobacteria](https://www.ncbi.nlm.nih.gov/Taxonomy/Browser/wwwtax.cgi?id=1236) | 68.9 | [1](https://blast.ncbi.nlm.nih.gov/Blast.cgi) |
| ..[Roseicyclus sp.](https://www.ncbi.nlm.nih.gov/Taxonomy/Browser/wwwtax.cgi?id=1914329) | [a-proteobacteria](https://www.ncbi.nlm.nih.gov/Taxonomy/Browser/wwwtax.cgi?id=28211) | 67.4 | [3](https://blast.ncbi.nlm.nih.gov/Blast.cgi) |
| ..[Oceanospirillum multiglobuliferum](https://www.ncbi.nlm.nih.gov/Taxonomy/Browser/wwwtax.cgi?id=64969) | [g-proteobacteria](https://www.ncbi.nlm.nih.gov/Taxonomy/Browser/wwwtax.cgi?id=1236) | 66.6 | [3](https://blast.ncbi.nlm.nih.gov/Blast.cgi) |
| ..[Aeromonas encheleia](https://www.ncbi.nlm.nih.gov/Taxonomy/Browser/wwwtax.cgi?id=73010) | [g-proteobacteria](https://www.ncbi.nlm.nih.gov/Taxonomy/Browser/wwwtax.cgi?id=1236) | 63.5 | [4](https://blast.ncbi.nlm.nih.gov/Blast.cgi) |
| ..[Aeromonas sp. sif2416](https://www.ncbi.nlm.nih.gov/Taxonomy/Browser/wwwtax.cgi?id=2854793) | [g-proteobacteria](https://www.ncbi.nlm.nih.gov/Taxonomy/Browser/wwwtax.cgi?id=1236) | 63.5 | [2](https://blast.ncbi.nlm.nih.gov/Blast.cgi) |
| ..[Aeromonas sp. sia0103](https://www.ncbi.nlm.nih.gov/Taxonomy/Browser/wwwtax.cgi?id=2854782) | [g-proteobacteria](https://www.ncbi.nlm.nih.gov/Taxonomy/Browser/wwwtax.cgi?id=1236) | 63.5 | [2](https://blast.ncbi.nlm.nih.gov/Blast.cgi) |
| ..[Maritalea sp. P4.10X](https://www.ncbi.nlm.nih.gov/Taxonomy/Browser/wwwtax.cgi?id=2909667) | [a-proteobacteria](https://www.ncbi.nlm.nih.gov/Taxonomy/Browser/wwwtax.cgi?id=28211) | 63.5 | [2](https://blast.ncbi.nlm.nih.gov/Blast.cgi) |
| ..[Burkholderia pseudomallei 1026b](https://www.ncbi.nlm.nih.gov/Taxonomy/Browser/wwwtax.cgi?id=884204) | [b-proteobacteria](https://www.ncbi.nlm.nih.gov/Taxonomy/Browser/wwwtax.cgi?id=28216) | 61.6 | [1](https://blast.ncbi.nlm.nih.gov/Blast.cgi) |
| ..[Burkholderia pseudomallei HBPUB10303a](https://www.ncbi.nlm.nih.gov/Taxonomy/Browser/wwwtax.cgi?id=1306419) | [b-proteobacteria](https://www.ncbi.nlm.nih.gov/Taxonomy/Browser/wwwtax.cgi?id=28216) | 61.6 | [1](https://blast.ncbi.nlm.nih.gov/Blast.cgi) |
| ..[Burkholderia pseudomallei 1026a](https://www.ncbi.nlm.nih.gov/Taxonomy/Browser/wwwtax.cgi?id=1085027) | [b-proteobacteria](https://www.ncbi.nlm.nih.gov/Taxonomy/Browser/wwwtax.cgi?id=28216) | 61.6 | [1](https://blast.ncbi.nlm.nih.gov/Blast.cgi) |
| ..[Burkholderia sp. 136(2017)](https://www.ncbi.nlm.nih.gov/Taxonomy/Browser/wwwtax.cgi?id=2020484) | [b-proteobacteria](https://www.ncbi.nlm.nih.gov/Taxonomy/Browser/wwwtax.cgi?id=28216) | 61.6 | [1](https://blast.ncbi.nlm.nih.gov/Blast.cgi) |
| ..[Burkholderia sp. 129](https://www.ncbi.nlm.nih.gov/Taxonomy/Browser/wwwtax.cgi?id=2020482) | [b-proteobacteria](https://www.ncbi.nlm.nih.gov/Taxonomy/Browser/wwwtax.cgi?id=28216) | 61.6 | [1](https://blast.ncbi.nlm.nih.gov/Blast.cgi) |
| ..[Burkholderia sp. 117](https://www.ncbi.nlm.nih.gov/Taxonomy/Browser/wwwtax.cgi?id=2020481) | [b-proteobacteria](https://www.ncbi.nlm.nih.gov/Taxonomy/Browser/wwwtax.cgi?id=28216) | 61.6 | [1](https://blast.ncbi.nlm.nih.gov/Blast.cgi) |
| ..[Burkholderia sp. 137](https://www.ncbi.nlm.nih.gov/Taxonomy/Browser/wwwtax.cgi?id=2020483) | [b-proteobacteria](https://www.ncbi.nlm.nih.gov/Taxonomy/Browser/wwwtax.cgi?id=28216) | 61.6 | [1](https://blast.ncbi.nlm.nih.gov/Blast.cgi) |
| ..[Burkholderia pseudomallei ABCPW 91](https://www.ncbi.nlm.nih.gov/Taxonomy/Browser/wwwtax.cgi?id=1434209) | [b-proteobacteria](https://www.ncbi.nlm.nih.gov/Taxonomy/Browser/wwwtax.cgi?id=28216) | 61.2 | [1](https://blast.ncbi.nlm.nih.gov/Blast.cgi) |
| ..[Burkholderia pseudomallei 576](https://www.ncbi.nlm.nih.gov/Taxonomy/Browser/wwwtax.cgi?id=557724) | [b-proteobacteria](https://www.ncbi.nlm.nih.gov/Taxonomy/Browser/wwwtax.cgi?id=28216) | 61.2 | [1](https://blast.ncbi.nlm.nih.gov/Blast.cgi) |
| ..[Burkholderia pseudomallei MSHR5855](https://www.ncbi.nlm.nih.gov/Taxonomy/Browser/wwwtax.cgi?id=1306421) | [b-proteobacteria](https://www.ncbi.nlm.nih.gov/Taxonomy/Browser/wwwtax.cgi?id=28216) | 61.2 | [1](https://blast.ncbi.nlm.nih.gov/Blast.cgi) |
| ..[Burkholderia pseudomallei MSHR5848](https://www.ncbi.nlm.nih.gov/Taxonomy/Browser/wwwtax.cgi?id=1306420) | [b-proteobacteria](https://www.ncbi.nlm.nih.gov/Taxonomy/Browser/wwwtax.cgi?id=28216) | 61.2 | [1](https://blast.ncbi.nlm.nih.gov/Blast.cgi) |
| ..[Burkholderia pseudomallei MSHR62](https://www.ncbi.nlm.nih.gov/Taxonomy/Browser/wwwtax.cgi?id=1435984) | [b-proteobacteria](https://www.ncbi.nlm.nih.gov/Taxonomy/Browser/wwwtax.cgi?id=28216) | 61.2 | [1](https://blast.ncbi.nlm.nih.gov/Blast.cgi) |
| ..[Burkholderia pseudomallei B03](https://www.ncbi.nlm.nih.gov/Taxonomy/Browser/wwwtax.cgi?id=1439854) | [b-proteobacteria](https://www.ncbi.nlm.nih.gov/Taxonomy/Browser/wwwtax.cgi?id=28216) | 61.2 | [1](https://blast.ncbi.nlm.nih.gov/Blast.cgi) |
| ..[Burkholderia pseudomallei A79A](https://www.ncbi.nlm.nih.gov/Taxonomy/Browser/wwwtax.cgi?id=1439855) | [b-proteobacteria](https://www.ncbi.nlm.nih.gov/Taxonomy/Browser/wwwtax.cgi?id=28216) | 61.2 | [1](https://blast.ncbi.nlm.nih.gov/Blast.cgi) |
| ..[Burkholderia pseudomallei 1258a](https://www.ncbi.nlm.nih.gov/Taxonomy/Browser/wwwtax.cgi?id=1086032) | [b-proteobacteria](https://www.ncbi.nlm.nih.gov/Taxonomy/Browser/wwwtax.cgi?id=28216) | 61.2 | [1](https://blast.ncbi.nlm.nih.gov/Blast.cgi) |
| ..[Burkholderia pseudomallei 1258b](https://www.ncbi.nlm.nih.gov/Taxonomy/Browser/wwwtax.cgi?id=1086033) | [b-proteobacteria](https://www.ncbi.nlm.nih.gov/Taxonomy/Browser/wwwtax.cgi?id=28216) | 61.2 | [1](https://blast.ncbi.nlm.nih.gov/Blast.cgi) |
| ..[Burkholderia pseudomallei MSHR1043](https://www.ncbi.nlm.nih.gov/Taxonomy/Browser/wwwtax.cgi?id=1247713) | [b-proteobacteria](https://www.ncbi.nlm.nih.gov/Taxonomy/Browser/wwwtax.cgi?id=28216) | 61.2 | [1](https://blast.ncbi.nlm.nih.gov/Blast.cgi) |
| ..[Burkholderia pseudomallei MSHR6137](https://www.ncbi.nlm.nih.gov/Taxonomy/Browser/wwwtax.cgi?id=1408264) | [b-proteobacteria](https://www.ncbi.nlm.nih.gov/Taxonomy/Browser/wwwtax.cgi?id=28216) | 61.2 | [1](https://blast.ncbi.nlm.nih.gov/Blast.cgi) |
| ..[Burkholderia pseudomallei MSHR5613](https://www.ncbi.nlm.nih.gov/Taxonomy/Browser/wwwtax.cgi?id=1435050) | [b-proteobacteria](https://www.ncbi.nlm.nih.gov/Taxonomy/Browser/wwwtax.cgi?id=28216) | 61.2 | [1](https://blast.ncbi.nlm.nih.gov/Blast.cgi) |
| ..[Burkholderia pseudomallei MSHR4868](https://www.ncbi.nlm.nih.gov/Taxonomy/Browser/wwwtax.cgi?id=1435363) | [b-proteobacteria](https://www.ncbi.nlm.nih.gov/Taxonomy/Browser/wwwtax.cgi?id=28216) | 61.2 | [1](https://blast.ncbi.nlm.nih.gov/Blast.cgi) |
| ..[Burkholderia pseudomallei MSHR465J](https://www.ncbi.nlm.nih.gov/Taxonomy/Browser/wwwtax.cgi?id=1435987) | [b-proteobacteria](https://www.ncbi.nlm.nih.gov/Taxonomy/Browser/wwwtax.cgi?id=28216) | 61.2 | [1](https://blast.ncbi.nlm.nih.gov/Blast.cgi) |
| ..[Burkholderia pseudomallei MSHR4377](https://www.ncbi.nlm.nih.gov/Taxonomy/Browser/wwwtax.cgi?id=1434200) | [b-proteobacteria](https://www.ncbi.nlm.nih.gov/Taxonomy/Browser/wwwtax.cgi?id=28216) | 61.2 | [1](https://blast.ncbi.nlm.nih.gov/Blast.cgi) |
| ..[Burkholderia pseudomallei MSHR4372](https://www.ncbi.nlm.nih.gov/Taxonomy/Browser/wwwtax.cgi?id=1434198) | [b-proteobacteria](https://www.ncbi.nlm.nih.gov/Taxonomy/Browser/wwwtax.cgi?id=28216) | 61.2 | [1](https://blast.ncbi.nlm.nih.gov/Blast.cgi) |
| ..[Burkholderia pseudomallei MSHR4503](https://www.ncbi.nlm.nih.gov/Taxonomy/Browser/wwwtax.cgi?id=1434206) | [b-proteobacteria](https://www.ncbi.nlm.nih.gov/Taxonomy/Browser/wwwtax.cgi?id=28216) | 61.2 | [1](https://blast.ncbi.nlm.nih.gov/Blast.cgi) |
| ..[Burkholderia pseudomallei TSV 43](https://www.ncbi.nlm.nih.gov/Taxonomy/Browser/wwwtax.cgi?id=1434202) | [b-proteobacteria](https://www.ncbi.nlm.nih.gov/Taxonomy/Browser/wwwtax.cgi?id=28216) | 61.2 | [1](https://blast.ncbi.nlm.nih.gov/Blast.cgi) |
| ..[Burkholderia pseudomallei MSHR4300](https://www.ncbi.nlm.nih.gov/Taxonomy/Browser/wwwtax.cgi?id=1434194) | [b-proteobacteria](https://www.ncbi.nlm.nih.gov/Taxonomy/Browser/wwwtax.cgi?id=28216) | 61.2 | [1](https://blast.ncbi.nlm.nih.gov/Blast.cgi) |
| ..[Burkholderia pseudomallei TSV 31](https://www.ncbi.nlm.nih.gov/Taxonomy/Browser/wwwtax.cgi?id=1434204) | [b-proteobacteria](https://www.ncbi.nlm.nih.gov/Taxonomy/Browser/wwwtax.cgi?id=28216) | 61.2 | [1](https://blast.ncbi.nlm.nih.gov/Blast.cgi) |
| ..[Burkholderia pseudomallei MSHR4003](https://www.ncbi.nlm.nih.gov/Taxonomy/Browser/wwwtax.cgi?id=1435369) | [b-proteobacteria](https://www.ncbi.nlm.nih.gov/Taxonomy/Browser/wwwtax.cgi?id=28216) | 61.2 | [1](https://blast.ncbi.nlm.nih.gov/Blast.cgi) |
| ..[Burkholderia pseudomallei BDU 2](https://www.ncbi.nlm.nih.gov/Taxonomy/Browser/wwwtax.cgi?id=1434211) | [b-proteobacteria](https://www.ncbi.nlm.nih.gov/Taxonomy/Browser/wwwtax.cgi?id=28216) | 61.2 | [1](https://blast.ncbi.nlm.nih.gov/Blast.cgi) |
| ..[Burkholderia pseudomallei MSHR4375](https://www.ncbi.nlm.nih.gov/Taxonomy/Browser/wwwtax.cgi?id=1434199) | [b-proteobacteria](https://www.ncbi.nlm.nih.gov/Taxonomy/Browser/wwwtax.cgi?id=28216) | 61.2 | [1](https://blast.ncbi.nlm.nih.gov/Blast.cgi) |
| ..[Burkholderia pseudomallei MSHR3964](https://www.ncbi.nlm.nih.gov/Taxonomy/Browser/wwwtax.cgi?id=1434927) | [b-proteobacteria](https://www.ncbi.nlm.nih.gov/Taxonomy/Browser/wwwtax.cgi?id=28216) | 61.2 | [1](https://blast.ncbi.nlm.nih.gov/Blast.cgi) |
| ..[Burkholderia pseudomallei MSHR3951](https://www.ncbi.nlm.nih.gov/Taxonomy/Browser/wwwtax.cgi?id=1434192) | [b-proteobacteria](https://www.ncbi.nlm.nih.gov/Taxonomy/Browser/wwwtax.cgi?id=28216) | 61.2 | [1](https://blast.ncbi.nlm.nih.gov/Blast.cgi) |
| ..[Burkholderia pseudomallei MSHR3960](https://www.ncbi.nlm.nih.gov/Taxonomy/Browser/wwwtax.cgi?id=1434203) | [b-proteobacteria](https://www.ncbi.nlm.nih.gov/Taxonomy/Browser/wwwtax.cgi?id=28216) | 61.2 | [1](https://blast.ncbi.nlm.nih.gov/Blast.cgi) |
| ..[Burkholderia pseudomallei MSHR1357](https://www.ncbi.nlm.nih.gov/Taxonomy/Browser/wwwtax.cgi?id=1435992) | [b-proteobacteria](https://www.ncbi.nlm.nih.gov/Taxonomy/Browser/wwwtax.cgi?id=28216) | 61.2 | [1](https://blast.ncbi.nlm.nih.gov/Blast.cgi) |
| ..[Burkholderia pseudomallei TSV5](https://www.ncbi.nlm.nih.gov/Taxonomy/Browser/wwwtax.cgi?id=1439861) | [b-proteobacteria](https://www.ncbi.nlm.nih.gov/Taxonomy/Browser/wwwtax.cgi?id=28216) | 61.2 | [1](https://blast.ncbi.nlm.nih.gov/Blast.cgi) |
| ..[Burkholderia pseudomallei TSV28](https://www.ncbi.nlm.nih.gov/Taxonomy/Browser/wwwtax.cgi?id=1439860) | [b-proteobacteria](https://www.ncbi.nlm.nih.gov/Taxonomy/Browser/wwwtax.cgi?id=28216) | 61.2 | [1](https://blast.ncbi.nlm.nih.gov/Blast.cgi) |
| ..[Burkholderia pseudomallei A79D](https://www.ncbi.nlm.nih.gov/Taxonomy/Browser/wwwtax.cgi?id=1439857) | [b-proteobacteria](https://www.ncbi.nlm.nih.gov/Taxonomy/Browser/wwwtax.cgi?id=28216) | 61.2 | [1](https://blast.ncbi.nlm.nih.gov/Blast.cgi) |
| ..[Burkholderia pseudomallei A79C](https://www.ncbi.nlm.nih.gov/Taxonomy/Browser/wwwtax.cgi?id=1439856) | [b-proteobacteria](https://www.ncbi.nlm.nih.gov/Taxonomy/Browser/wwwtax.cgi?id=28216) | 61.2 | [1](https://blast.ncbi.nlm.nih.gov/Blast.cgi) |
| ..[Burkholderia pseudomallei MSHR1328](https://www.ncbi.nlm.nih.gov/Taxonomy/Browser/wwwtax.cgi?id=1249469) | [b-proteobacteria](https://www.ncbi.nlm.nih.gov/Taxonomy/Browser/wwwtax.cgi?id=28216) | 61.2 | [1](https://blast.ncbi.nlm.nih.gov/Blast.cgi) |
| ..[Neisseria canis](https://www.ncbi.nlm.nih.gov/Taxonomy/Browser/wwwtax.cgi?id=493) | [b-proteobacteria](https://www.ncbi.nlm.nih.gov/Taxonomy/Browser/wwwtax.cgi?id=28216) | 62.4 | [3](https://blast.ncbi.nlm.nih.gov/Blast.cgi) |
| .[Oscillatoria sp. HE19RPO](https://www.ncbi.nlm.nih.gov/Taxonomy/Browser/wwwtax.cgi?id=2954806) | [cyanobacteria](https://www.ncbi.nlm.nih.gov/Taxonomy/Browser/wwwtax.cgi?id=1117) | 85.5 | [1](https://blast.ncbi.nlm.nih.gov/Blast.cgi) |
| .[Prevotella sp.](https://www.ncbi.nlm.nih.gov/Taxonomy/Browser/wwwtax.cgi?id=59823) | [CFB group bacteria](https://www.ncbi.nlm.nih.gov/Taxonomy/Browser/wwwtax.cgi?id=976) | 82.8 | [1](https://blast.ncbi.nlm.nih.gov/Blast.cgi) |
| .[Candidatus Entotheonella factor](https://www.ncbi.nlm.nih.gov/Taxonomy/Browser/wwwtax.cgi?id=1429438) | [bacteria](https://www.ncbi.nlm.nih.gov/Taxonomy/Browser/wwwtax.cgi?id=-1) | 78.6 | [1](https://blast.ncbi.nlm.nih.gov/Blast.cgi) |
| .[Bacteroidales](https://www.ncbi.nlm.nih.gov/Taxonomy/Browser/wwwtax.cgi?id=171549) | [CFB group bacteria](https://www.ncbi.nlm.nih.gov/Taxonomy/Browser/wwwtax.cgi?id=976) | 79.0 | [1](https://blast.ncbi.nlm.nih.gov/Blast.cgi) |
| .[Bacteroides thetaiotaomicron VPI-5482](https://www.ncbi.nlm.nih.gov/Taxonomy/Browser/wwwtax.cgi?id=226186) | [CFB group bacteria](https://www.ncbi.nlm.nih.gov/Taxonomy/Browser/wwwtax.cgi?id=976) | 79.0 | [10](https://blast.ncbi.nlm.nih.gov/Blast.cgi) |
| .[Bacteroides thetaiotaomicron](https://www.ncbi.nlm.nih.gov/Taxonomy/Browser/wwwtax.cgi?id=818) | [CFB group bacteria](https://www.ncbi.nlm.nih.gov/Taxonomy/Browser/wwwtax.cgi?id=976) | 79.0 | [19](https://blast.ncbi.nlm.nih.gov/Blast.cgi) |
| .[Cyanomargarita calcarea GSE-NOS-MK-12-04C](https://www.ncbi.nlm.nih.gov/Taxonomy/Browser/wwwtax.cgi?id=2839659) | [cyanobacteria](https://www.ncbi.nlm.nih.gov/Taxonomy/Browser/wwwtax.cgi?id=1117) | 77.8 | [1](https://blast.ncbi.nlm.nih.gov/Blast.cgi) |
| .[Bacteroides faecis](https://www.ncbi.nlm.nih.gov/Taxonomy/Browser/wwwtax.cgi?id=674529) | [CFB group bacteria](https://www.ncbi.nlm.nih.gov/Taxonomy/Browser/wwwtax.cgi?id=976) | 74.3 | [14](https://blast.ncbi.nlm.nih.gov/Blast.cgi) |
| .[Cytophagales bacterium](https://www.ncbi.nlm.nih.gov/Taxonomy/Browser/wwwtax.cgi?id=2053541) | [CFB group bacteria](https://www.ncbi.nlm.nih.gov/Taxonomy/Browser/wwwtax.cgi?id=976) | 73.9 | [3](https://blast.ncbi.nlm.nih.gov/Blast.cgi) |
| .[Bacteroides sp. CAG:754](https://www.ncbi.nlm.nih.gov/Taxonomy/Browser/wwwtax.cgi?id=1262750) | [CFB group bacteria](https://www.ncbi.nlm.nih.gov/Taxonomy/Browser/wwwtax.cgi?id=976) | 73.9 | [1](https://blast.ncbi.nlm.nih.gov/Blast.cgi) |
| .[Gemmatimonadetes bacterium](https://www.ncbi.nlm.nih.gov/Taxonomy/Browser/wwwtax.cgi?id=2026742) | [bacteria](https://www.ncbi.nlm.nih.gov/Taxonomy/Browser/wwwtax.cgi?id=-1) | 73.2 | [1](https://blast.ncbi.nlm.nih.gov/Blast.cgi) |
| .[Bacteroides congonensis](https://www.ncbi.nlm.nih.gov/Taxonomy/Browser/wwwtax.cgi?id=1871006) | [CFB group bacteria](https://www.ncbi.nlm.nih.gov/Taxonomy/Browser/wwwtax.cgi?id=976) | 72.4 | [3](https://blast.ncbi.nlm.nih.gov/Blast.cgi) |
| .[Phocaeicola vulgatus](https://www.ncbi.nlm.nih.gov/Taxonomy/Browser/wwwtax.cgi?id=821) | [CFB group bacteria](https://www.ncbi.nlm.nih.gov/Taxonomy/Browser/wwwtax.cgi?id=976) | 72.0 | [12](https://blast.ncbi.nlm.nih.gov/Blast.cgi) |
| .[Muribaculaceae bacterium](https://www.ncbi.nlm.nih.gov/Taxonomy/Browser/wwwtax.cgi?id=2498093) | [CFB group bacteria](https://www.ncbi.nlm.nih.gov/Taxonomy/Browser/wwwtax.cgi?id=976) | 69.3 | [1](https://blast.ncbi.nlm.nih.gov/Blast.cgi) |
| .[Lachnospiraceae bacterium](https://www.ncbi.nlm.nih.gov/Taxonomy/Browser/wwwtax.cgi?id=1898203) | [firmicutes](https://www.ncbi.nlm.nih.gov/Taxonomy/Browser/wwwtax.cgi?id=1239) | 68.9 | [1](https://blast.ncbi.nlm.nih.gov/Blast.cgi) |
| .[Prevotella copri](https://www.ncbi.nlm.nih.gov/Taxonomy/Browser/wwwtax.cgi?id=165179) | [CFB group bacteria](https://www.ncbi.nlm.nih.gov/Taxonomy/Browser/wwwtax.cgi?id=976) | 68.9 | [6](https://blast.ncbi.nlm.nih.gov/Blast.cgi) |
| .[Lachnoclostridium sp.](https://www.ncbi.nlm.nih.gov/Taxonomy/Browser/wwwtax.cgi?id=2028282) | [firmicutes](https://www.ncbi.nlm.nih.gov/Taxonomy/Browser/wwwtax.cgi?id=1239) | 67.4 | [1](https://blast.ncbi.nlm.nih.gov/Blast.cgi) |
| .[Blautia sp.](https://www.ncbi.nlm.nih.gov/Taxonomy/Browser/wwwtax.cgi?id=1955243) | [firmicutes](https://www.ncbi.nlm.nih.gov/Taxonomy/Browser/wwwtax.cgi?id=1239) | 67.4 | [1](https://blast.ncbi.nlm.nih.gov/Blast.cgi) |
| .[Bacteroides thetaiotaomicron dnLKV9](https://www.ncbi.nlm.nih.gov/Taxonomy/Browser/wwwtax.cgi?id=1235785) | [CFB group bacteria](https://www.ncbi.nlm.nih.gov/Taxonomy/Browser/wwwtax.cgi?id=976) | 67.4 | [1](https://blast.ncbi.nlm.nih.gov/Blast.cgi) |
| .[Chloroherpeton thalassium](https://www.ncbi.nlm.nih.gov/Taxonomy/Browser/wwwtax.cgi?id=100716) | [green sulfur bacteria](https://www.ncbi.nlm.nih.gov/Taxonomy/Browser/wwwtax.cgi?id=1090) | 65.1 | [1](https://blast.ncbi.nlm.nih.gov/Blast.cgi) |
| .[Chloroherpeton thalassium ATCC 35110](https://www.ncbi.nlm.nih.gov/Taxonomy/Browser/wwwtax.cgi?id=517418) | [green sulfur bacteria](https://www.ncbi.nlm.nih.gov/Taxonomy/Browser/wwwtax.cgi?id=1090) | 65.1 | [1](https://blast.ncbi.nlm.nih.gov/Blast.cgi) |
| .[Microcystis aeruginosa LG13-13](https://www.ncbi.nlm.nih.gov/Taxonomy/Browser/wwwtax.cgi?id=2685981) | [cyanobacteria](https://www.ncbi.nlm.nih.gov/Taxonomy/Browser/wwwtax.cgi?id=1117) | 63.9 | [1](https://blast.ncbi.nlm.nih.gov/Blast.cgi) |
| .[Microcystis aeruginosa LG13-03](https://www.ncbi.nlm.nih.gov/Taxonomy/Browser/wwwtax.cgi?id=2685978) | [cyanobacteria](https://www.ncbi.nlm.nih.gov/Taxonomy/Browser/wwwtax.cgi?id=1117) | 63.9 | [1](https://blast.ncbi.nlm.nih.gov/Blast.cgi) |
| .[Microcystis aeruginosa LG11-05](https://www.ncbi.nlm.nih.gov/Taxonomy/Browser/wwwtax.cgi?id=2685977) | [cyanobacteria](https://www.ncbi.nlm.nih.gov/Taxonomy/Browser/wwwtax.cgi?id=1117) | 63.9 | [1](https://blast.ncbi.nlm.nih.gov/Blast.cgi) |
| .[Microcystis wesenbergii TW10](https://www.ncbi.nlm.nih.gov/Taxonomy/Browser/wwwtax.cgi?id=2060474) | [cyanobacteria](https://www.ncbi.nlm.nih.gov/Taxonomy/Browser/wwwtax.cgi?id=1117) | 63.9 | [1](https://blast.ncbi.nlm.nih.gov/Blast.cgi) |
| .[Chitinophagales bacterium](https://www.ncbi.nlm.nih.gov/Taxonomy/Browser/wwwtax.cgi?id=2448779) | [CFB group bacteria](https://www.ncbi.nlm.nih.gov/Taxonomy/Browser/wwwtax.cgi?id=976) | 63.5 | [1](https://blast.ncbi.nlm.nih.gov/Blast.cgi) |
| .[Chitinophagaceae bacterium](https://www.ncbi.nlm.nih.gov/Taxonomy/Browser/wwwtax.cgi?id=1869212) | [CFB group bacteria](https://www.ncbi.nlm.nih.gov/Taxonomy/Browser/wwwtax.cgi?id=976) | 62.4 | [1](https://blast.ncbi.nlm.nih.gov/Blast.cgi) |
| .[Rikenellaceae bacterium](https://www.ncbi.nlm.nih.gov/Taxonomy/Browser/wwwtax.cgi?id=2049048) | [CFB group bacteria](https://www.ncbi.nlm.nih.gov/Taxonomy/Browser/wwwtax.cgi?id=976) | 62.4 | [1](https://blast.ncbi.nlm.nih.gov/Blast.cgi) |
| .[Tenacibaculum litoreum](https://www.ncbi.nlm.nih.gov/Taxonomy/Browser/wwwtax.cgi?id=321269) | [CFB group bacteria](https://www.ncbi.nlm.nih.gov/Taxonomy/Browser/wwwtax.cgi?id=976) | 62.0 | [2](https://blast.ncbi.nlm.nih.gov/Blast.cgi) |
| .[Parabacteroides pacaensis](https://www.ncbi.nlm.nih.gov/Taxonomy/Browser/wwwtax.cgi?id=2086575) | [CFB group bacteria](https://www.ncbi.nlm.nih.gov/Taxonomy/Browser/wwwtax.cgi?id=976) | 60.8 | [1](https://blast.ncbi.nlm.nih.gov/Blast.cgi) |
| .[Tenacibaculum caenipelagi](https://www.ncbi.nlm.nih.gov/Taxonomy/Browser/wwwtax.cgi?id=1325435) | [CFB group bacteria](https://www.ncbi.nlm.nih.gov/Taxonomy/Browser/wwwtax.cgi?id=976) | 62.0 | [2](https://blast.ncbi.nlm.nih.gov/Blast.cgi) |
| .[Aquimarina sp. AD10](https://www.ncbi.nlm.nih.gov/Taxonomy/Browser/wwwtax.cgi?id=1714849) | [CFB group bacteria](https://www.ncbi.nlm.nih.gov/Taxonomy/Browser/wwwtax.cgi?id=976) | 62.0 | [3](https://blast.ncbi.nlm.nih.gov/Blast.cgi) |

**Supplementary Table S4.** Taxonomy report for an BLASTP search of the GenBank nr protein database limited to exclude Eukaryota (taxid:2759) with putative zebrafish (*Danio rerio*) thiaminase I sequence [NP_001314821.1](https://www.ncbi.nlm.nih.gov/sites/entrez?cmd=Search&db=protein&term=NP_001314821.1&dopt=GenBank), Max target sequences = 100.

| Parameter | Default | Adjusted for short input sequences |
| --- | --- | --- |
| Max target sequences | 100 | 100 |
| Expect threshold | 0.05 | 200000 |
| Word Size | 6 | 2 |
| Max matches in a query range | 0 | 0 |
| Matrix | BLOSUM62 | PAM30 |
| Gap Costs | Existence: 11 Extension: 1 | Existence: 9 Extension: 1 |
| Compositional adjustments | Conditional compositional score matrix adjustment | No adjustment |
| Filters and Masking | Mask low complexity regions | No masking |

**Supplementary Table S5.** Summary of default BLASTP algorithm parameters and adjusted parameters for short input sequences.
